# Extraction of Nanochitin from Marine Resources and Fabrication of Polymer Nanocomposites: Recent Advances

**DOI:** 10.3390/polym12081664

**Published:** 2020-07-27

**Authors:** Blessy Joseph, Rubie Mavelil Sam, Preetha Balakrishnan, Hanna J. Maria, Sreeraj Gopi, Tatiana Volova, Susana C. M. Fernandes, Sabu Thomas

**Affiliations:** 1 International and Inter University Centre for Nanoscience and Nanotechnology, Mahatma Gandhi University, Kottayam, Kerala 686560, India; blessyprince14@gmail.com (B.J.); b.preethabalakrishnan@gmail.com (P.B.); hannavidhu@gmail.com (H.J.M.); 2 Research and Post Graduate Department of Chemistry, Bishop Moore College, Mavelikara, Kerala 690110, India; rubiemsams@gmail.com; 3 Plant Lipids Pvt. Ltd., Cochin, Kerala 682311, India; 4 Institute of Biophysics of Russian Academy of Science, Siberian Federal University, 660041 Krasnoyarsk, Russia; 5 Institute of Interdisciplinary Research on Environment and Materials (IPREM), Université de Pau et des Pays de l’Adour, E2S UPPA, CNRS, IPREM, 64600 Anglet, France; 6 School of Energy Materials, Mahatma Gandhi University, Kottayam, Kerala 686560, India

**Keywords:** nanochitin, biodegradable, marine, reinforcement, polysaccharides

## Abstract

Industrial sea food residues, mainly crab and shrimp shells, are considered to be the most promising and abundant source of chitin. In-depth understanding of the biological properties of chitin and scientific advancements in the field of nanotechnology have enabled the development of high-performance chitin nanomaterials. Nanoscale chitin is of great economic value as an efficient functional and reinforcement material for a wide range of applications ranging from water purification to tissue engineering. The use of polymers and nanochitin to produce (bio) nanocomposites offers a good opportunity to prepare bioplastic materials with enhanced functional and structural properties. Most processes for nanochitin isolation rely on the use of chemical, physical or mechanical methods. Chitin-based nanocomposites are fabricated by various methods, involving electrospinning, freeze drying, etc. This review discusses the progress and new developments in the isolation and physico-chemical characterization of chitin; it also highlights the processing of nanochitin in various composite and functional materials.

## 1. Introduction

Recently, several studies focused on developing value-added products from marine resources, providing a large number of biomaterials that are of economic importance. Marine organisms synthesize a variety of biopolymers having interesting and unique functional, structural and biological properties with multifunctional applications in different industrial sectors. They are mainly classified into three categories: polysaccharides, proteins and nucleic acids [1]. In particular, polysaccharides—alginate, chitosan, chitin, etc.—are used for multifunctional applications owing to their biodegradability and non-toxic potential. Marine biopolymers find great potential in theranostic applications owing to their stability and biocompatibility. They can be easily surface-modified due to the presence of hydroxyl, carboxyl and amino groups, which makes them preferred candidates for drug delivery applications [2].

**Figure 1:**
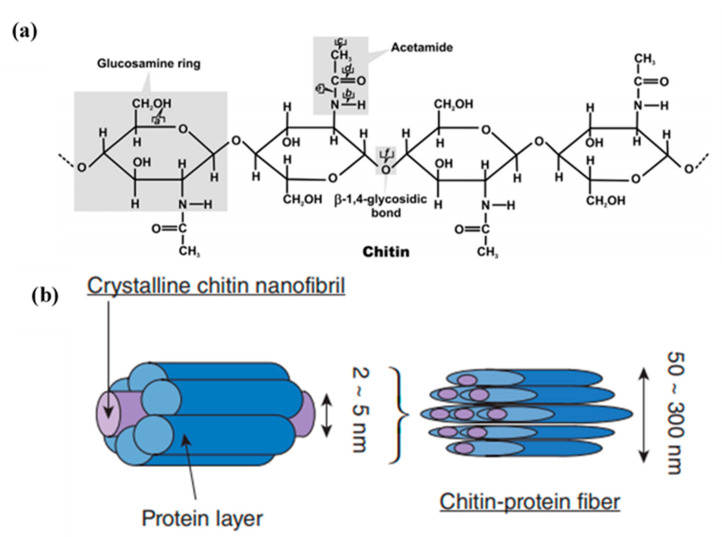
(**a**) Chemical structure of chitin [4]. (**b**) Representation of native chitin nanofibrils in crustacean shells [5].

Chitin, in particular, is a linear polysaccharide composed of *N*-acetyl-2-amido-2-deoxy-d-glucose units linked by β(1→4) bonds (Figure 1a) and is considered the second most abundant biopolymer on earth after cellulose [3]. 

It is the main structural component of crab, lobster and shrimp exoskeletons. The crustaceous shells are mainly made up of chitin (20–30%), proteins (30–40%), calcium carbonate (30–50%), lipids and astaxanthin (less than 1%). The crustacean shells present a complex hierarchical organization. In crustacean shells, chitin macromolecules are the basic units that combine together to form chitin nanofibers. They occur as highly ordered crystalline microfibrils with strong intermolecular and intramolecular hydrogen bonding. Chitin has been known to form microfibrillar arrangements (2–5 nm) and long lengths (several μm) embedded in a protein matrix, forming chitin protein fibers with diameters ranging from 50 to 300 nm (Figure 1b). Then, this woven network of planes, wherein chitin is embedded in proteins and calcium carbonate, forms twisted or helicoidal stacking sequences, called a Bouligand structure [4,6]. These chitin micro fibrils can be isolated and used as relevant reinforcements or functional agents in composite materials, as is discussed all in this review article. 

**Figure 2:**
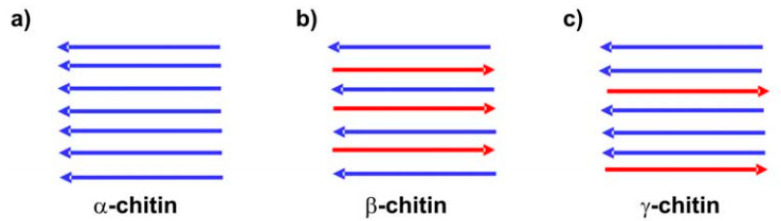
Three polymorphic configurations of chitin: (**a**) α-chitin, (**b**) β-chitin, (**c**) γ-chitin [8].

Three allomorphs of chitin exist, namely, α, β and γ-chitin. The crystalline structures of α-chitin and β-chitin can be elucidated from the electron diffraction patterns of highly crystalline samples. There are two antiparallel chains per unit cell in α-chitin, contrary to β-chitin, which has a parallel arrangement. In γ-chitin two chains are arranged in one direction and the other chain in the opposite direction (Figure 2). α-Chitin possess strong inter and intra sheet hydrogen bonding, whereas β-chitin has weak intra-sheet hydrogen bonding [7]. 

Due to its highly ordered crystalline structure, chitin is resistant to physical and chemical agents. Chitin is insoluble in most common solvents. This insolubility seriously limits the development and application of chitin. Chitosan, a well-known derivative of chitin, is formed through the enzymatic or chemical deacetylation of chitin. Chitosan is also a copolymer of β-(1→4)-*N*-acetyl-d-glucosamine and β-(1→4)-d-glucosamine [9]. The degree of acetylation (DA) helps to define chitin and chitosan. DA represents the proportion of *N*-acetyl-D-glucosamine units with respect to the total number of units. When the degree of deacetylation (DDA) of chitin reaches about 50%, the product is named chitosan and becomes soluble in acidic aqueous solutions [10]. Due to its biocompatibility, biodegradability and bioactivity, chitosan has several applications in waste-water treatment, agriculture, cosmetics and food processing [11].

Chitin is biodegradable, and due to its biodegradable nature, it could find immense applications in packaging and composite materials. Due to the presence of chitinases and chitinase-like proteins in the human body, chitin has been used in surgical sutures, tissue engineering and drug delivery [12,13,14]. Chitin and chitin derivatives can stimulate immune cells and they also show significant anticancer activity [15,16,17,18]. The effect of chitin against fungal disease candidiasis has also been reported [19]. Chitin nanofibrils and nanocrystals have shown UV-protection ability, a moisturizing effect and anti-aging properties and are used in skin protective formulations [20,21]. 

In order to meet the growing demand for chitin due to its unique properties, there has been profound interest in transforming the shellfish food industry’s waste (mainly crab and shrimp) into useful products. Amid the COVID-19 crisis, the global market for chitin and chitosan derivatives estimated at 106.9 thousand metric tons in the year 2020, is projected to reach a revised size of 281.7 thousand metric tons by 2027, following a report from Global Industry Analysts Inc.,USA. Japan and USA are the leading producers of chitin, followed by India, Italy and Poland [22]. About 40–55% in the case of shrimp and over 70% in the case of crabs are discarded as waste during the transformation processing. Depending on the processing method, the waste of these species contains approximately between 10% and 55% chitin on a dry weight basis. 

To make it more viable for practical applications, in the last 30 years, research has been directed towards nanoscale chitin materials. Nanochitin has a larger surface area than corresponding bulk materials, which favors the filler–matrix interactions, thereby resulting in enhanced performance of the final composite material. In this context, this review complies recent advances in nanochitin isolation and its use in composite materials.

## 2. Extraction of Nanochitin

**Figure 3:**
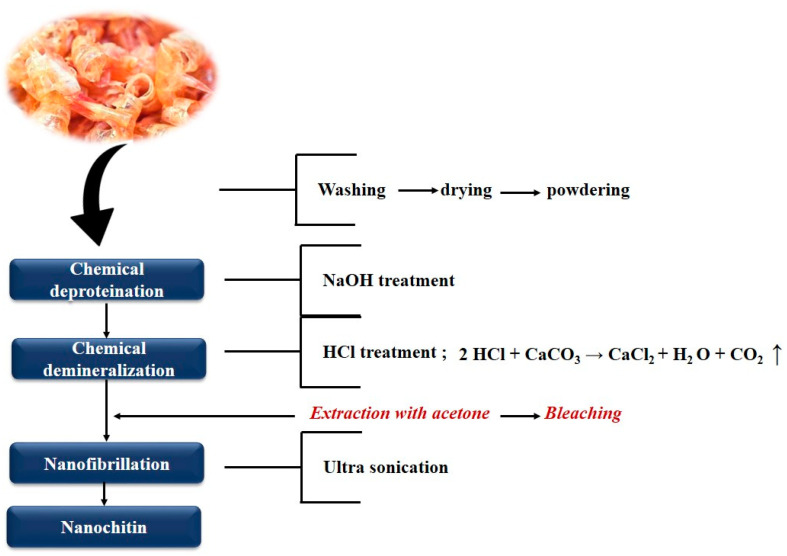
General protocol for extracting chitin nanofibers from prawn shells by using ultra-sonication.

The extraction of chitin nanofibers or nanocrystals from the complex hierarchy of crustaceans’ exoskeletons involves various steps. They include the preparation of raw chitin, including chitin extraction via the demineralization and deproteinization of shrimp, crab or lobster shell wastes; and finally, the isolation of nanochitin. Substantial efforts have been made to develop chemical, mechanical and enzymatic methods to obtain purified products. Initially, the matrix components such as protein and calcium carbonate are removed by treating the crustacean’s shells with NaOH and HCl aqueous solutions, respectively. In actual practice the extraction of chitin from shellfish involves the step-by-step removal of two major constituents of the shell, the intimately associated proteins by deproteinization and inorganic calcium carbonate by demineralization [23]. The pigments and lipids are removed by extraction with ethanol or acetone after the demineralization process with dilute hydrochloric acid at room temperature. A wide range of chemicals have been tried as deproteinization reagents, including NaOH, Na_2_CO_3_, NaHCO_3_, KOH, K_2_CO_3_, Ca (OH)_2_, Na_2_SO_3_, NaHSO_3_, CaHSO_3_, Na_3_PO_4_ and Na_2_S. NaOH is the preferred reagent on the basis of its performance and typically a 1M NaOH solution is used with variations in the temperature and duration of treatment parameters. The use of NaOH invariably results in partial deacetylation of chitin and hydrolysis of the biopolymer that lowers the molecular weight of chitin [24,25]. Normally the residual protein content in the chitin produced from conventional commercial sources is around 1%. This yields a partially deacetylated chitin. The obtained chitin flakes or powder can be further deacetylated to chitosan or used for the isolation of nanochitin: chitin nanowhiskers (or nanocrystals) and chitin nanofibers. As already mentioned, herein, we focus on recent advances in nanochitin isolation and its use on composite materials. As example, the schematic representation of steps involved in isolation of chitin nanofibers via ultra-sonication is shown in Figure 3. 

### 2.1. Chitin Nanowhiskers (CNW)

Similarly to the preparation of cellulose nanowhiskers, the main process for isolation of chitin nanowhiskers from extracted chitin is also based on acid hydrolysis. Disordered and low lateral ordered regions of chitin are preferentially hydrolyzed and dissolved in the acid solution, whereas water-insoluble, highly crystalline residues that have higher resistance to acid attack remain intact [26]. Thus, following acid hydrolysis, which removes disordered and low lateral ordered crystalline defects, chitin rod-like whiskers are obtained. The swelling and hydrolysis of amorphous phases occur much faster than those of crystalline phases due to the regular tight arrangement of molecular chains in the crystalline domains. It is well established and documented that the boiling hydrochloric acid can easily dissolve the amorphous domains of chitin. 

When chitin is treated with strong HCl acid, the amorphous structures will be dissolved completely, but over hydrolysis, the ether and amide linkages of chitin can be affected. Thus, it is essential to control the hydrolytic extent of chitin to obtain a good yield of chitin nanowhiskers with desirable size. The overall factors affecting the yield and particle size of a chitin nanowhiskers are the (1) origin of chitin, (2) concentration of HCl and (3) hydrolytic time. Thus, different conditions have been studied taking in account the different chitin origins [27]. If the concentration of acid increases, the crystalline structures will be gradually damaged, and for concentration of 8.5 N or higher, the crystalline structures will be completely dissolved. When comparing the studies conducted by various groups on the preparation of CNW, it was observed that for 3 N HCl concentration, the hydrolytic time (from 1.5 to 6 h) did not affect the size, especially the cross-sectional width of the nanowhisker, which showed similar lateral dimensions.

Another significant method used for chitin nanowhiskers’ isolation is the called TEMPO: mediated oxidation (2, 2, 6, 6-tetramethylpiperidine-1-oxyl radical mediated oxidation). The oxidation of purified chitin takes place in the presence of compounds such as NaBr and NaClO with a pH of 10. After oxidation reactions, the major products remaining will be water-soluble polyuronic acid and water insoluble particles. Reaction rates of these two products can be controlled by the amount of NaClO in the system. The water-insoluble particles are the whiskers, which will undergo further ultra-sonication treatment. By this process, very narrow particles (width < 10 nm) can be obtained with a good yield rate of approximately 90% [27,28].

Based on these approaches, whiskers have recently been prepared from chitins of different sources, such as crab shells, shrimp shells, lobster shells and squid pens [29,30,31,32,33,34,35,36].

Lu et al. [36], reported for the first time a route for preparing suspensions of chitin crystallite particles in 1993. In this method, purified chitin was first treated within a 2.5 N hydrochloric acid solution under reflux for 1 h; the excess acid was decanted and then distilled water was added to obtain the suspension. They found that acid-hydrolyzed chitin spontaneously dispersed into rod-like particles that could be concentrated to a liquid crystalline phase and self-assemble to a cholesteric liquid crystalline phase above a certain concentration. Dufresne and co-workers have successfully isolated the crystalline regions of chitin whiskers from the crab shells and squid pens by hydrochloric acid hydrolysis [29,30]. The crystallites obtained were rod-like particles with an average size of 200 ± 20 nm in length and 8 ± 1 nm in width. Because of the nanoscale size, the acicular crystals can be called nanocrystals or whiskers. Morin and Dufresne [37] also prepared nanochitin whiskers from Riftia (marine invertebrates). The diameter of those whiskers was 18 nm and lengths were around 120 nm. In another study, Gopalan and Dufresne extracted nanochitin whiskers from crab shell. They successfully extracted 100–600 nm in length and 4–40 nm in width nanocrystals form 500–1000 µm chitin microcrystals [29]. 

Rujiravanit and co-workers reported the preparation of chitin whiskers by acid hydrolysis of shrimp shells [38]. The nanochitin whiskers consisted of slender rods with sharp points that had a broad distribution in terms of size. The length of the chitin fragments ranged from 150 to 800 nm; the width ranged from 5 to 70 nm. More than 75% of the whiskers, however, had a length below 420 nm. Lu and co-workers prepared nanochitin whiskers from crab shell. They were spindle shaped with broad distribution in length (L), ranging from 100 to 650 nm, and diameter (D), ranging from 10 to 80 nm. The averages of length and diameter were estimated to be 500 and 10 nm, respectively. Revol et al. and Li et al. reported an approach toward the preparation of a suspension of chitin crystallites through acid hydrolysis [39,40]. The obtained crystallites in a colloidal state were rod like particles with average size of 200 ± 20 nm in length and 8 ± 1 nm in width. Salaberria et al. extracted chitin from yellow lobster wastes followed by the isolation of nanocrystals by acid hydrolysis [41]. The ensuing chitin nanocrystals presented a random rod-like morphology with average diameter of 60 nm and length of 300 nm.

### 2.2. Chitin Nanofibrils (CNF) or Chitin Nanofibers

Since chitin occurs in nature in the form of nanofibrillar networks, it is possible to obtain chitin nanofibers from prawn, crab, shrimp and lobster shells and squid pen chitin by mechanical and physical processing. Those kinds of processes favor transverse cleavage along the longitudinal axis of the chitin microfibrillar structure by high shear, resulting in the isolation of long nanochitin fibers. Blender grinding; high-pressure homogenizing; and combinations of those and other techniques with ultrasonic treatments have been used [42,43,44,45]. Typically, nanofibers have a great aspect ratio; a high surface to volume ratio; and contrarily to chitin nanocrystals, which present mainly crystal domains, chitin nanofibers present amorphous and crystal regions. Chitin nanofibers are very long fibers with a diameter ranging from 10 to 20 nm, giving a high aspect ratio of approximately 100 [45]. Moreover, nanofibers generally possess unique mechanical, optical and other characteristics, which allow them to be used in different fields. 

Grinder equipment has been used to disintegrate the aggregated chitin nanofibers, and in this process, an aqueous suspension of chitin usually 1 wt % of concentration will be passed through the specially designed grinding stones. After the grinding process, the slurry will be formed into gel, which is well dispersed into water [43]. This method of manufacturing is applicable for many varieties of prawns, such as Penaeus monodon (black tiger prawn), Marsupenaeus japonicas (Japanese tiger prawn), and Pandalus eous Makarov (Alaskan pink shrimp). These shrimp are commonly used in the food industry and widely cultured around the globe [46]. Salaberria et al. used a dynamic high-pressure homogenization to obtain chitin nanofibers from yellow lobster [44]. This homogenization technique is based on the passage of a chitin suspension (1 wt % in water) at a very high pressure through a homogenizing valve. This passage is able to downsize chitin into chitin nanofibers. With this approach, the authors obtained chitin nanofibers with diameters below 100 nm and several micrometers in length.

## 3. Properties of Nanochitin

### 3.1. Physico-Chemical Properties

A number of studies have been reported in the literature about the chemical structure of chitin by using infrared spectra. [31,47,48] Usually, the C=O stretching region of the amide moiety is found between 1600 and 1500 cm^−1^; the sharp band at 1378 cm^−1^ is assigned to CH_3_ symmetrical deformation. The band at 1656 cm^−1^ is commonly assigned to stretching of the C=O group hydrogen bonded to N–H of the neighboring intra-sheet chain. The band at 1621 cm^−1^ may indicate a specific hydrogen bond of C=O with the hydroxymethyl group of the next chitin residue of the same chain. This is reinforced by the presence of only one band in this region for *N*-acetyl d-glucosamine [24]. In α-chitin, the amide I band is split at 1660 and 1620 cm^−1^ and the amide II band is at 1556 cm^−1^. The amide I absorption band is a single peak at 1650 cm^−1^ for β-chitin, whereas the amide I bands for γ-chitin are at 1660 cm^−1^ and 1620 cm^−1^. 

**Figure 4:**
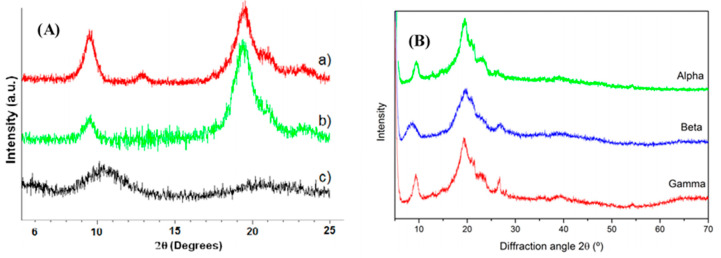
(**A**) XRD spectra of (**a**) chitin flakes, (**b**) chitin nanofibrils (CNF) and (**c**) a chitosan nanoparticles and chitosan nanofibers mixture [49]; and (**B**) X-ray diffractograms α, β and γ-chitin [50].

In X-ray diffraction measurement, the sample is bombarded with X-rays and the diffraction pattern produced is recorded. In general, broad, diffuse and less intense peaks are observed for chitosan, and for chitin well-resolved and intense peaks are observed, showing the more crystalline nature of chitin compared to chitosan. Figure 4A shows the XRD spectra of chitin flakes, chitin nanofibrils and chitosan NPs and chitosan [49]. 

In the case of chitin and nanochitin, four major diffraction peaks located around 9.5°, 19.5°, 20.9° and 23.4 corresponding to the 0 2 0, 1 1 0, 1 2 0, 1 3 0 crystal planes could be observed. However, for chitosan the two main diffraction peaks are located at 10°–11° and at 20°–21°. The peak shows broad diffraction peaks, indicating an amorphous polymer structure compared to chitin. This change is explained as the distortion of macromolecular configuration induced by strong deacetylation conditions. As previously discussed, depending on its origin, chitin can occur in three crystalline forms, α, β and γ-forms. Compared to α-chitin, β-chitin exhibits a broad diffuse scattering and less intense peaks. This is due to the differences in the crystallographic arrangements of these two polymorphs [51]. Kaya et al. analyzed the diffraction patterns in chitin. Six crystalline reflection peaks were obtained at 2-theta range (Figure 4B). I_020_ crystalline reflection values were at 9.46, 8.59° and 9.35° for α, β and γ-chitin, respectively [50]. The crystalline index (CrI) value of γ-chitin was 68.6° and was between that of α and β-chitin.

### 3.2. Morphology of Nanochitin

**Figure 5:**
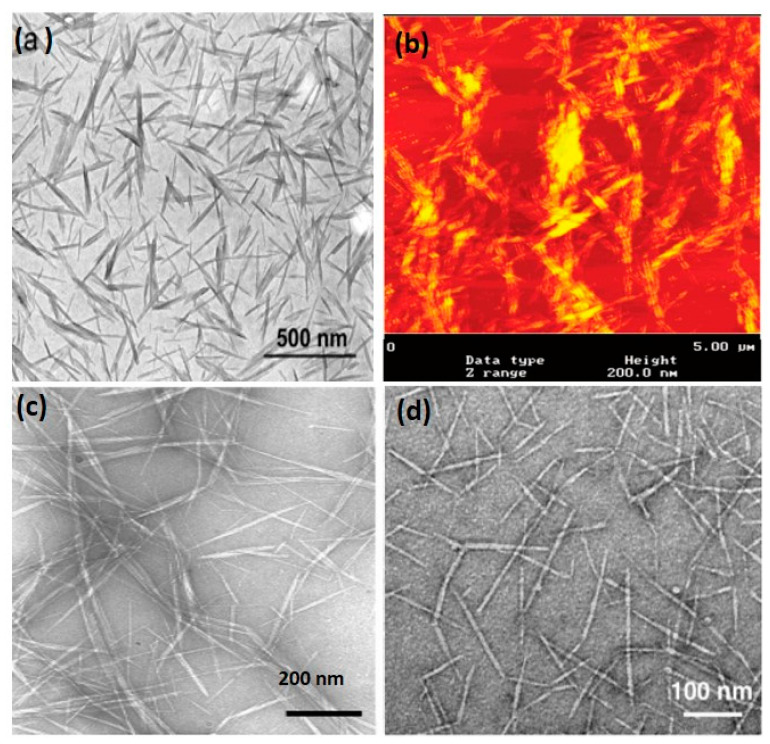
(**a**) TEM [30] and (**b**) AFM [36] images of a dilute suspension of chitin whiskers; (**c**) TEM images of individual chitin whiskers obtained by TEMPO method [28] and (**d**) TEM pictures of individual chitin whiskers from α-chitin partially-deacetylated by fibril surface cationization [54].

Following acid hydrolysis that removes disordered and low lateral ordered crystalline defects, chitin rod-like whiskers are obtained [52]. The principle toward preparation of polysaccharide nanocrystals is on the basis of the different hydrolytic kinetics between amorphous and crystalline domains [53]. That is to say, both the amorphous and the crystalline phases of polysaccharides can be hydrolyzed during treatment with strong acid aqueous solutions. Unlike tunicin whiskers, which can only be prepared by hydrolysis in strong sulfuric acid solutions, CNWs can be prepared by hydrolysis in HCl solutions. In general, the obtained rod-like whiskers showed similar size in width in the range of 10−50 nm, irrespective of the chitin’s origins and hydrolytic time. However, the lengths of the whiskers greatly vary in the range of 150−2200 nm for different origins of chitin, which may be ascribed to the different original sizes of the chitin particles and the diffusion-controlled nature of the acid hydrolysis. Typical morphologies and sizes (average length and width) of dilute CHW suspension observed by TEM and AFM are displayed in Figure 5a,b [29,36].

The suspension contains chitin fragments consisting of both individual microcrystals and associated or collapsed microcrystals. More recently, individual chitin nanowhiskers have been prepared from partially deacetylated α-chitin by fibril surface cationization. When CNWs with degrees of *N*-acetylation (DA) from 0.74 to 0.70 were mechanically disintegrated in aqueous solutions from pH 3 to 4, individualized nanowhiskers of average width and length 6.2 ± 1.1 and 250 ± 140 nm were successfully prepared, as reported by Fan et al. [54] The driving force for individualization of CNWs is the same as that in the TEMPO-mediated oxidation method, that is, surface charge-induced electrical repulsion. The differences in the two methods are that TEMPO-mediated oxidation endows CNW surfaces with anionic charges versus cationic charges for the latter. The TEM image and sizes of the individual CNWs are shown in Figure 4c,d; some long individual fibrils, with widths similar to those of the whiskers, but lengths of >500 nm, were observed, which have not been reported in previous CNWs prepared by the conventional method. 

The morphology of α-chitin nanofibers isolated by dynamic, high-pressure homogenization is quite different from α-chitin isolated by acid hydrolysis and TEMPO. It consists of long chitin nanofibers presenting lengths of several micrometers (from 3 to 10 μm) and widths between 80 and 100 nm and a high aspect ratio (greater than 60) [44].

## 4. Nanochitin-Based Polymer Nanocomposites

Numerous polymer composites have been produced from a variety of natural materials extracted from waste and biomass sources, and a plethora of processing techniques have also been studied. Production of novel polymer materials from nanochitin through physical methods relying extensively on green technology was found to be sustainable.

They are characterized by their excellent mechanical properties and reinforcing capability, abundance in availability, low weight and biodegradability. However, just as for any nanoparticle, the main challenge is related to their homogeneous dispersion within a polymeric matrix. Chitin nanocrystals have a reactive surface covered with hydroxyl groups, opening up the possibility of extensive chemical modification. Using surfactants or by chemical grafting/modifications, these nanocrystals can be dispersed in non-aqueous media. This strategy not only decreases the surface energy and polar character of the polysaccharide nanoparticles, but improves their adhesion property with a non-polar polymeric matrix, which imposes serious constraints when the mechanical performance of the composite is considered. The reinforcement effect shown by nanocrystals is generally due to the formation of a percolating network of hydrogen bond.

Different processing techniques exist for nanochitin-reinforced polymer nanocomposites wherein chitin nanocrystal fillers are blended with polymeric matrices, such as poly (methyl methacrylate), epoxy, polystyrene, polyaniline, polysulfone, polycarbonate and thermoplastic polyurethane [55]. Polymer based blends show incompatibility due to repulsion between polar nanochitin functional groups and hydrophobic polymeric matrices. Studies were done on the addition of chitin nanocrystals as a compatibilizer in a blend system. Additionally, nanochitin has been blended with hydrophilic and bio-based polymeric matrices, namely, chitosan, starch, PLA, cellulose nanocrystals [33,41,56,57,58,59]. There are many potential applications for polymer/nanochitin in membrane technology, dye removal, packaging materials, drug delivery, tissue engineering and biochemical relevance. Among the processing techniques for the development of nanochitin-reinforced polymer nanocomposites, freeze-drying, solvent casting, extrusion and electrospinning are described below.

### 4.1. Freeze-Drying

The freeze-drying approach, which allows producing aerogels having high porosity and internal surface area and low heat conductivity, is a widely accepted technique. The dispersion of nanoparticles in the nanocomposite suspension depends on the processing technique and conditions. 

**Figure 6:**
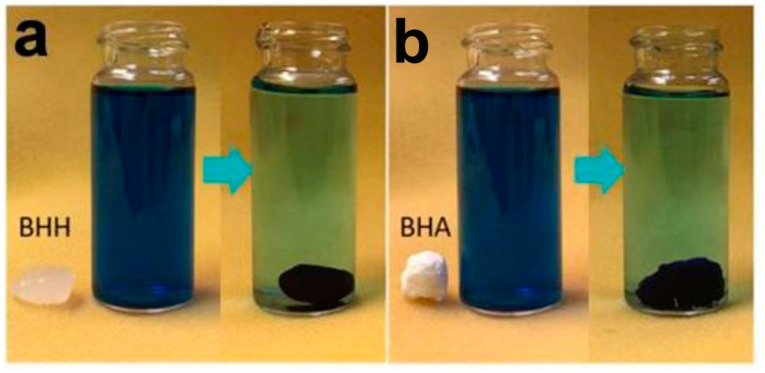
Digital image of methylene blue absorption by BHH (**a**) and BHA (**b**) [60].

Nanocellulose-nanochitin biohybrid aerogels (BHA) were produced by Zhang et al. by freeze drying hydrogels (BHH) [60]. The aerogels were found to exhibit adsorption potential for Arsenite and methylene blue (Figure 6). 

The mechanism behind the self-assembly of nanocellulose and nanochitin was found to be electrostatic interaction. Aerogels were produced from surface-modified chitin nanowhiskers and carbon nanotubes (CNTs). The resulting aerogels had decreased storage and loss modulus with the addition of CNT, which could be related to the change in morphology upon the incorporation of CNT [61]. Zubillaga et al. designed and made genipin–chitosan cross-linked matrices impregnated with chitin nanoforms by freeze-drying method. The potential of the obtained 3D porous scaffolds as primary support and guidance for stem cells in tissue engineering and regenerative medicine were assessed. In vitro cell biocompatibility studies showed that both L-929 and human adipose stem cells were viable in contact with the extractive media of these biomaterials; and cells were able to adhere and proliferate on the 3D porous scaffolds. Moreover, the addition of chitin nanoforms improved cell adhesion at low chitin nanoform ratios [56]. More recently, the same authors assessed the adipose-derived mesenchymal stem cell chondrospheroids cultured in hypoxia in the same 3D porous scaffolds as a platform for cartilage tissue engineering [33].

### 4.2. Casting/Solvent Evaporation

**Figure 7:**
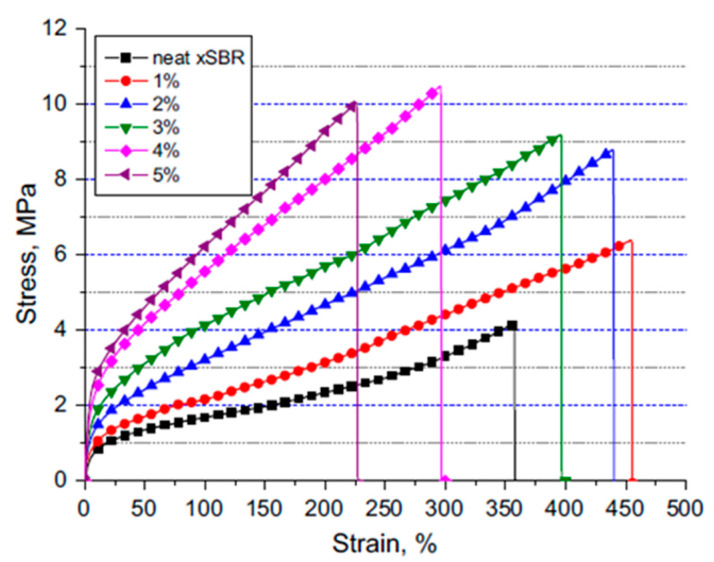
The tensile stress–strain curves for xSBR and xSBR/chitin nanocrystals nanocomposites [63].

Casting/solvent evaporation is another method of producing nanocomposites, wherein the polymer is first dissolved in an appropriate solvent (e.g., organic, aqueous) and chitin nanoparticles are blended into the polymer solution using different mixing techniques, namely, Ultra-Turrax, stirring and ultra-sound, to obtain homogenous dispersion. Afterwards, the suspension is cast in a mold, and finally the solvent is evaporated to get a solid nanocomposite film. Several investigations mentioned the use of water-soluble, water-dispersible polymers such as chitosan and starch [41,62]. Carboxylated styrene-butadiene rubber (xSBR) composites were prepared with chitin nanocrystals using the solvent casting method [63]. The chitin nanocrystals proved to improve the mechanical properties of the composite in terms of elastic modulus, tensile strength and strain at break of xSBR, as seen from the stress–strain graph given in Figure 7.

This study shows that uniform distribution of nanofillers in a polymer matrix could drastically influence the overall properties of a composite. 

### 4.3. Extrusion

The preparation of nanocomposites by melt extrusion is carried out by pumping the suspension of nanocrystals into the polymer melt during the extrusion process [64]. Chitin nano-size fillers were incorporated in thermoplastic starch matrix via melt-mixing [59]. The thermoplastic starch-based nano-bio composites prepared with chitin nanofibers showed better thermal and mechanical properties and storage moduli than those prepared with chitin nanocrystals.

Polypropylene (PP)/chitin nanowhisker composites showed increase in tensile properties, which is common with addition of nanofillers [65]. The compatibilizer used was maleated PP. There was no significant change in rheological properties. Moreover, the properties decreased with nanofillers’ incorporation as the material became rigid. This can be explained by the fact that at low filler addition, the mobility of the matrix is restrained, and at higher addition, loading agglomeration occurs, resulting in stress-concentration sites. The case was different with polylactide (PLA) and chitin nanocomposites prepared by melt blending [66]. Due to the hydrophobic nature of PLA, the nanocomposites were also modified with compatibilizing agent maleic anhydride. Here, contrary to the above work, the tensile strengths of both nanocomposite and modified nanocomposites decreased with the addition of chitin due to hydrolysis of PLA during composite preparation. Rheology studies showed that addition of chitin decreases the elastic storage modulus.

### 4.4. Electrospinning

Electrospinning is a method used to prepare nanofibers with diameters up to 100 nm through the action of electrostatic forces. Here electrical charge is used to draw a positively charged polymer solution from an orifice to a collector [67]. The process is simple and does not require the use of high temperatures or chemical treatments to produce nanofibers from solution.

Junkasem et al. reported the fabrication of polyvinyl alcohol (PVA)/chitin nanowhisker nanocomposites using electrospinning with water as the solvent. The electrospun composite membranes exhibited bead formation, while neat PVA showed smooth morphology. The Young’s modulus of the nanocomposite also increased 4–8 times when compared to the pristine PVA [68]. Polyvinylidene fluoride (PVDF)/CNW membranes were fabricated by our group by employing a DMF:acetone solvent mixture. The membranes had good mechanical strength; moreover, due to hydrophilicity of the membranes water could pass through by diffusion retaining oil droplets on the surface, demonstrating its potential for oil-water separation [69]. Previously, we had reported similar membranes fabricated by electrospinning a 15% solution of PVDF with 1% CNW in dimethyl acetamide (DMAc); 88.9% removal of the dye indigo carmine could be achieved with neat PVDF showing only 22.3% removal alone, the governing mechanism being hydrogen bonding and the electrostatic force of attraction. The main highlight of this work was the reusability of the membranes for three cycles [24].

## 5. Characterization of Chitin Nanowhiskers Containing Polymer Composites

### 5.1. Transmission Electron Microscopy

For studying the properties of nanostructured materials at the nanometer scale, investigation of nanostructures is required. Transmission electron microscopy (TEM) is one of the best techniques used for the characterization of nanomaterials and nanocomposites. It gives a precise idea of nanoparticle size; grain size; size distribution; homogeneity; lattice type; crystal structure; dispersion; and also the chemical and physical properties of phases such as number, morphology and structure of each phase. It provides structural and chemical information over a range of length scales down to the level of atomic dimensions. TEM is an unavoidable tool for understanding the properties of nanostructured materials.

**Figure 8:**
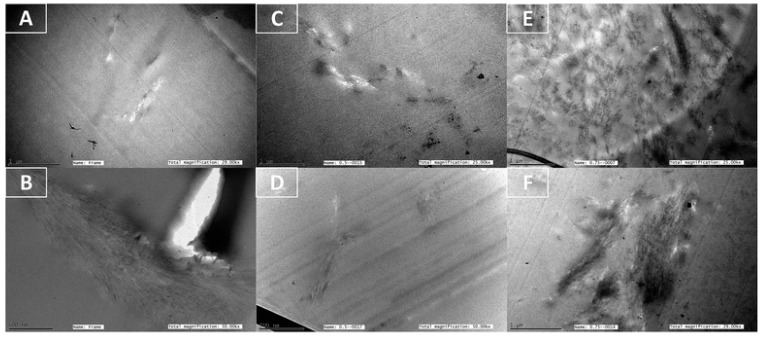
TEM micrographs of epoxy CNW nanocomposites. (**A**,**B**) 0.25 wt % CNWs, (**C**,**D**) 0.5 wt % CNWs, and (**E**,**F**) 0.75 wt % CNWs. The sections were negatively stained with uranyl acetate [70]. Scale bar A, C, E and F-1 μm, B and D-500 nm.

Anwer et al. in their work on biodegradable bionanocomposites evaluated the effect of CNWs as reinforcing agents in epoxy. They studied the morphological properties of DGEBA epoxy. The TEM images could confirm the presence of CNW clusters within the epoxy matrix [70] (Figure 8). 

**Figure 9:**
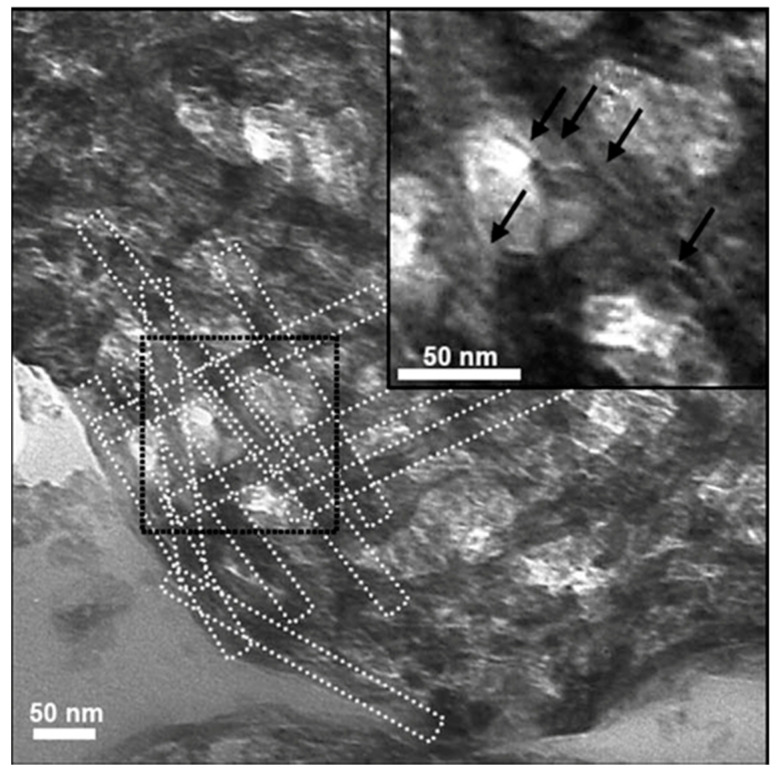
TEM analysis of spray-dried microparticles obtained with high initial chitin volume fractions [71]. The sample is formed by entangled rods of silica (white dashed rectangles) separated by voids (10–100 nm). These rods come from the initial chitin nanorods represented by the rectangles (23 × 260 nm^2^). Inside the silica rods, the imprint of the chitin monocrystals (2–3 nm wide) can be distinguished (black arrows in the zoomed area).

In a study reported by Alomnso et al., the chitin–silica nanocomposites and mesoporous materials were prepared by sol–gel processes through a colloid-based approach using elongated chitin nanorods [71]. They could observe a spheroid shape of spray-dried chitin-silica particles from the TEM analysis. The result suggests that the chitin nano rods were coated by siloxane oligomers and formed hybrid rods (Figure 9). The TEM image showed silica rods of 2–3 nm wide, and the imprint of the chitin monocrystals (marked in the image).

### 5.2. Scanning Electron Microscopy (SEM) 

The use of different characterization techniques is important to understand the basic physical and chemical properties of polymer nanocomposites. For several applications, it facilitates the study of emerging materials by giving information on intrinsic properties. Various techniques have been used extensively in polymer nanocomposite research. Structural and morphological characterization by scanning electron microscopy (SEM) uses a focused beam of electrons which provides images of the surface associated with a sample. It can be used to observe the fracture surfaces of polymer nanocomposites and to see the dispersion of nanoparticles in polymers across the failed surface. SEM is supportive in clarifying the presence of pores, impurities and morphological changes. SEM could also be supportive in explaining the dispersion of nanofiller materials.

**Figure 10:**
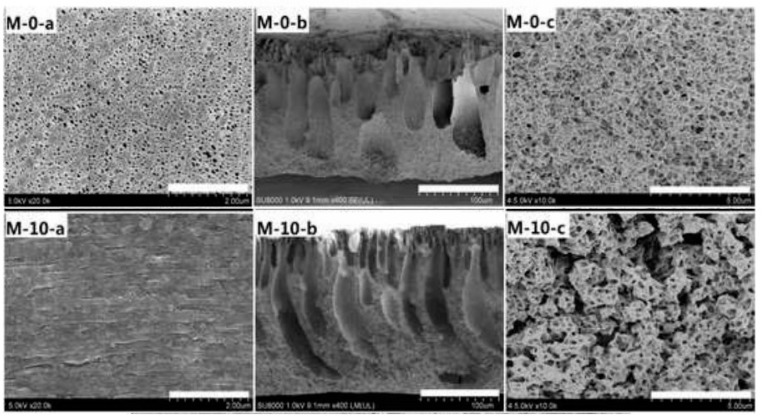
SEM images of PVDF/CNW composite membranes (**a**): surface; (**b**): cross-section; (**c**): enlargement of the rectangular area; (M-0-PVDF alone; M-10-PVDF + 10 wt % CNW) [72].

For instance, in a study reported by Qin et al. PVDF-CNW membranes were prepared with 18 wt % PVDF and 0–10 wt % of CNW. They observed that the average surface pore size of composite membranes was comparatively smaller than that of neat PVDF membrane, with the pores becoming more uniform with further addition chitin nanowhiskers. This explains the uniform dispersion of chitin nanowhiskers on PVDF matrix, as seen from Figure 10 [72].

The effect of increasing chitin nanowhisker content leading to the formation of an interconnected structure in membrane could be explained using the SEM. This morphological development was due to the tendency of chitin nanowhiskers to adsorb water, resulting in a liquid–liquid phase separation. The adhesion between different materials and the propagation of applied stress in a composite system can be explained with the support of SEM images.

**Figure 11:**
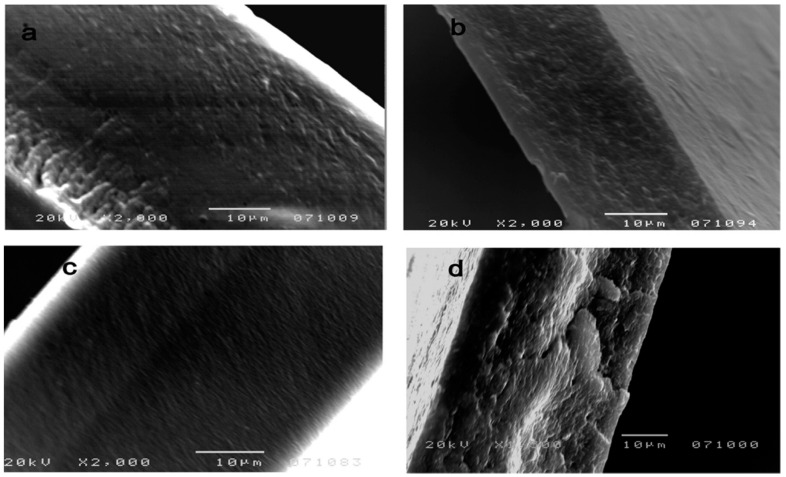
Scanning electron microscopy images of (**a**) uncrosslinked chitosan, (**b**) uncrosslinked chitosan–chitin nanocrystals composite, (**c**) crosslinked chitosan and (**d**) crosslinked chitosan–chitin nanocrystals [73].

In a work by Mathew et al. the fracture surfaces of the uncross-linked and cross-linked chitosan/chitin crystal nanocomposites did not show any large agglomerates, indicating good adhesion between the matrix and the reinforcement, and the fracture propagated through the matrix rather than through the chitosan–chitin interface [73] (Figure 11).

**Figure 12:**
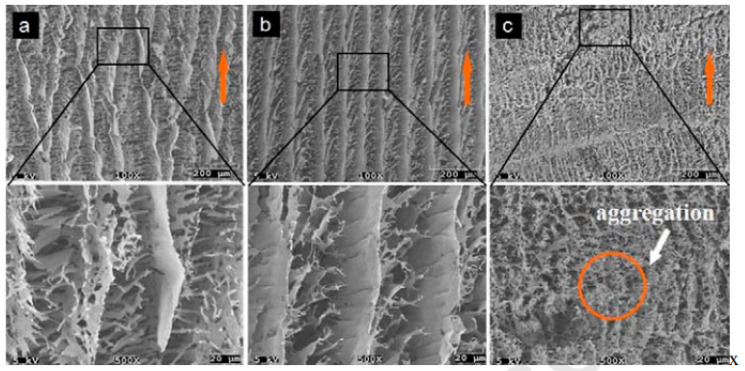
SEM images of CNW foams made with the various CNW suspensions under directional freezing. The suspension concentrations were (**a**) 0.4 wt %, (**b**) 0.8 wt %, (**c**) 1.2 wt %. Red arrows indicate the freezing directions [74].

Porous foams were fabricated with PVA and chitin nanowhiskers using the freeze-drying technique [74]. PVA concentration was kept constant as 0.8 wt % and CNW concentrations were varied from 0.4–1.2 wt %. The changes in morphology with varying concentrations of CNW were analyzed by SEM, as shown in Figure 12. 

At 0.4 wt % CNW, several structural deformities could be seen with crack formation (Figure 12a). At 0.8 wt % of CNW, highly ordered lamellar structures with no evident defects were seen, as shown in Figure 12b. At higher concentrations, aggregations were seen (Figure 12c).

### 5.3. Mechanical Properties of Nanochitin Composites

To understand the mechanical properties, it is important to have a thorough knowledge of the chemistry and morphology of the polymer matrix and how it correlates with the surface chemistry, the size and the shape of a nanoscale filler. The chemistry of the nanoscale filler influences enthalpic interactions with the polymer chain, and specific interactions, such as covalent bonds. The effects of the mechanical properties of the filler on the overall mechanical properties of the nanocomposites have been explored by several research groups. They reported that for any nanofiller the mechanical properties depend on parameters including nanofiller size and shape, interfacial region, processing conditions, method of manufacture, etc. The interactions between the polymer matrix and nanofiller and filler–filler interactions influence the mechanical behavior of the polysaccharide, nanocrystal-reinforced nanocomposites. The adhesion of polysaccharide nanoparticles with a non-polar polymeric matrix is reported to cause a negative effect on the mechanical performances of the nanocomposite because of the formation of a percolating network through hydrogen bonding forces. 

A biodegradable porous scaffold of thermoset elastomer was fabricated by Tian et al., for which chitin-nanocrystal-supported emulsion-freeze-casting was utilized [75]. The mass percentages of chitin nanocrystals were controlled at 15%, 20%, 25%, 30%, 35% and 40%. The nanocomposite exhibited good elastic resilience and improved mechanical properties with increasing chitin nanocrystal content. In another report by Mushi et al., chitin nanofibers and chitosan-based biocomposite films of high toughness was prepared and characterized [76]. This biopolymer nanocomposite containing chitin nanofiber networks in chitosan matrix had the highest toughness at 8 vol. % chitin content, and at very high chitin content the nanocomposites showed tensile strength of 140 MPa, and strain to failure 11%. Nie et al. produced epoxidized natural rubber (ENR)/chitin nanocrystals composites without using conventional crosslinking agents. They explained that chitin nanocrystals reacted with the epoxy group of ENR and formed hydrogen bonds resulting in the formation of supramolecular network. This structure could effectively enhance the mechanical properties along with superior self-healing capacity [77].

**Figure 13:**
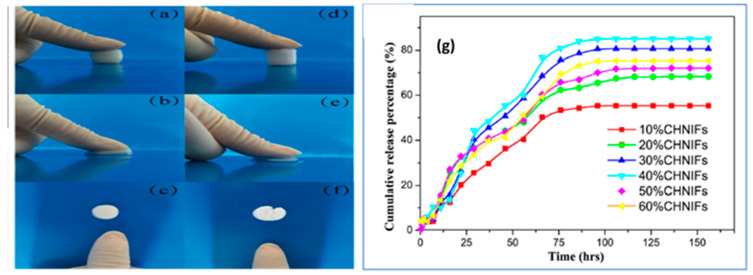
The pictures of PVA/CNW (30% CNWs, 1% GA) hydrogels (**a**–**c**) and pure PVA hydrogels (**d**–**f**) compressed by a thumb. (**g**) Release activities of BSA from the hydrogels at 25 °C [79].

The effect of chitin nanocrystals on the formation of shish-kebab crystals in bimodal polyethylene (BPE) injection bars was evaluated by Bie et al. [78]. They reported that the addition of chitin nanocrystals (0–0.5 wt % chitin nanocrystals) enhanced the ultimate tensile strength and Young’s modulus of the injection bars. Compared to BPE, the tensile modulus of composites with 0.3 wt % cellulose nanocrystals increased by 11.6%. The ability of chitin nanowhiskers to effectively act as a reinforcing filler was showed by Peng et al. using poly(vinyl alcohol) (PVA) and chitin nanowhiskers. PVA/CNW hydrogels for drug delivery applications were proposed with bovine serum albumin (BSA) as the model drug and glutaraldehyde as the crosslinker [79]. The mechanical properties were analyzed, and neat PVA displayed poor mechanical properties and was very fragile compared to PVA/CNWs hydrogel (Figure 13a–f).

However, the hydrogel comprised of PVA/CNWs was strong enough and the stress at fracture increased from 1.55 to 26.59 MPa with an increase in the ratio of CNWs to PVA content (0% to 40%). The drug release profile showed a release of more than 50% of drug within 60 h (Figure 13g).

### 5.4. Thermal Properties of Nanochitin Composites

The thermal properties of composites comprising of nanochitin have been analyzed by numerous researchers with the aid of thermo gravimetric analysis (TGA) and differential scanning calorimetry (DSC) [80,81,82] usually under a nitrogen atmosphere [80,83,84,85,86].

The thermal stability of carboxymethylcellulose (CMC)-based films containing chitin nanocrystals and grapefruit seed extract (GSE) was investigated by Oun et al. using thermogravimetric analysis under nitrogen flow; all films showed two stages of thermal degradation [84]. The preliminary phase was detected around 80–130 °C with weight loss extending from 4.9% to 7.9%, which was associated with the elimination of free water below 110 °C and bound water. The chief thermal degradation was observed at 200–400 °C, which was connected with the thermal degradation of carbohydrate polymers and the volatilization of glycerol. All films revealed analogous thermal degradation parameters of the initial decomposition temperature (T_onset_) in the range of 200–210 °C, the mid-point of the degradation (T_0.5_) of 270–275 °C and the end of degradation temperature (T_end_) of 321–333 °C. The final residue remaining after thermal degradation at 600 °C of the neat CMC film was 21.7%, but improved to about 30% thanks to the incorporation of nanocrystals and GSE.

**Figure 14:**
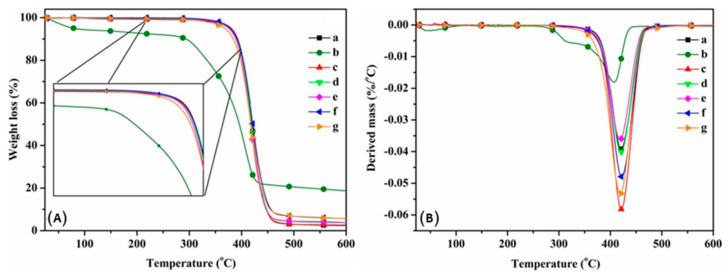
TGA (**A**) and DTG (**B**) of nanochitin sample and PBAT/chitin composites: (**a**) PBAT, (**b**) chitin, (**c**) chitin-0.5, (**d**) chitin-1, (**e**) chitin-2, (**f**) chitin-4 and (**g**) chitin-8 [80].

The thermal properties of biodegradable poly (butylene adipate-co-terephthalate) (PBAT) composites reinforced with bio-based nanochitin were investigated by Meng et al. using TGA and DSC under a nitrogen atmosphere [80]. TGA curves and derivative thermal and PBAT/nanochitin composites are shown in Figure 14a,b, respectively. 

**Figure 15:**
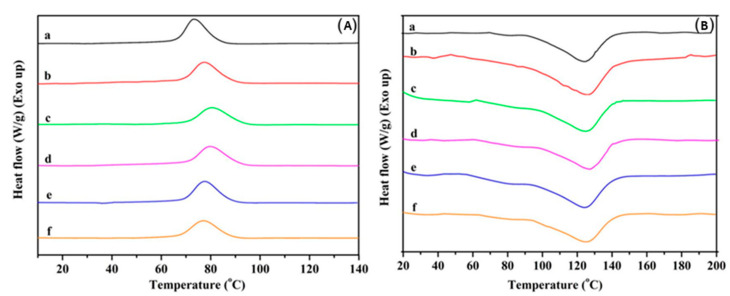
DSC cooling curves (**A**) and heating curves (**B**) for pristine PBAT and the different PBAT/chitin composites: (**a**) PBAT, (**b**) chitin-0.5, (**c**) chitin-1, (**d**) chitin-2, (**e**) chitin-4 and (**f**) chitin-8 [80].

The TGA curve for the nanochitin powder exhibited two discrete mass losses. The primary one happened below 100 °C and matches with the loss of physically absorbed water. The second and grander mass loss befell in the 280–400 °C range and could be ascribed to the carbonization of the polysaccharide structure. In contrast, the thermal degradation of pristine PBAT and the PBAT/nanochitin composites occurred in a one-step process at temperatures between 300 and 400 °C. The thermal stability of the PBAT/chitin composites was similar to pristine PBAT when the chitin content was lower than 4%. Char yields of the composites increased with chitin content, as expected since the chitin acted as an additional carbon source for char formation. Furthermore, the nanochitin delayed heat transmission along the PBAT matrix, consequently varying the carbonization kinetics of PBAT. DSC was used to examine the effect of nanochitin powder addition on the crystallization and melting behavior of PBAT. DSC cooling curves and second heating curves for pristine PBAT and the various PBAT/nanochitin composites are shown in Figure 15a,b, respectively.

**Figure 16:**
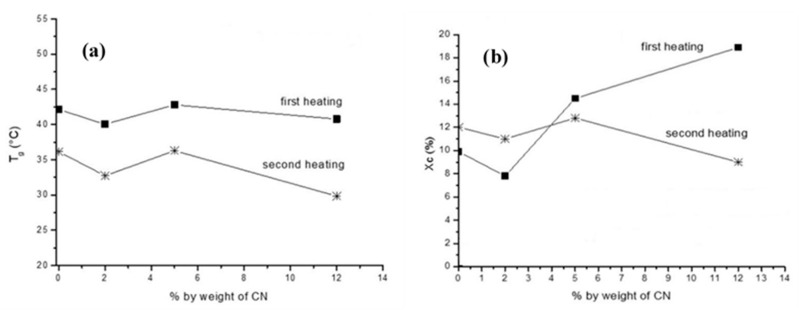
(**a**) Analysis of thermal properties from first and second heating steps in PLA/CNF nanocomposites: Trend of T_g_ as a function of CNF concentration. (**b**) Analysis of thermal properties from first and second heating steps in PLA/CNF nanocomposites: trend of crystallinity as a function of CNF concentration [81].

The addition of a small amount of nanochitin (0.5 wt %) had quite a dramatic effect of the melting and recrystallization of PBAT. The values of T_m_, ΔH_m_, T_c_, ΔH_c_ and χ_c_ for chitin-0.5 were all higher than the corresponding values for pristine PBAT. The data strongly recommend that integration of nanochitin at 0.5 wt % had a varied nucleation result, helping the establishment of crystallites in the PBAT matrix during cooling from the melt. The researchers concluded that the thermal properties of the composites were largely reliant on the concentration (0.5 wt % being optimal) and dispersion of nanochitin [80]. Coltelli et al., during their investigation on the thermal properties of poly (lactic acid) (PLA)-based composites comprising chitin nanofibrils using DSC under a nitrogen atmosphere, resolved that the concurrent existence of CNFs and triethyl citrate (TEC) in the studies composition range did not incite substantial deviations, apart from the apparent decline of the glass transition temperature owing to the plasticizing effect of TEC [81]. The inclinations of glass transition temperature (T_g_) and crystallinity (X_c_) as functions of CNF concentration (represented as CN in figure) are depicted in Figure 16a,b.

**Figure 17:**
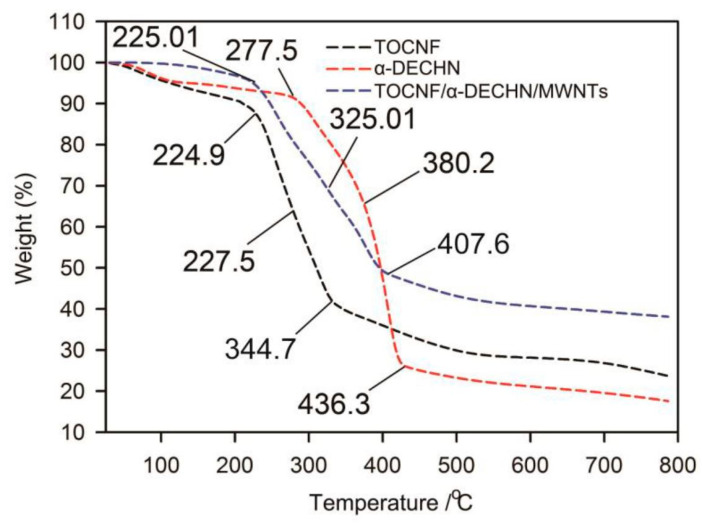
TGA profiles of TOCNF, α-DECHN, and TOCNF/MWCNT/α-DECHN [85].

The T_g_ values were practically unchanged, but higher for the first heating than for the second. The crystallinity X_c_ was almost constant as a function of CNF concentration in the second heating, displaying an insignificant consequence of CNFs on controlled crystallization. On the contrary, the crystallinity was pointedly amplified when the concentration of CNF was 5 and 12 wt %. As a consequence of CNF addition to the samples, the thermal properties exhibited few specific variations, only being responsible for a slight nucleating effect. This restructuring is apparent above 5 wt % of CNFs. In a study conducted by Xu et al., the compositions, thermal stabilities and decomposition kinetics of TEMPO-oxidized cellulose nanofiber (TOCNF), partially deacetylated α-chitin nanofiber (α-DECHN) and TOCNF/multi-wall carbon nanotube (MWCNT)/α-DECHN composite wires were analyzed by TGA [85]. As shown in the above Figure 17, the preliminary phase of the thermal decomposition of TOCNF/MWCNT/α-DECHN composite wire is consistent with that of TOCNFs.

As the temperature rises, the difference between the weight loss percentages of TOCNF/MWCNT/α-DECHN composite wire and α-DECHNs is steadily diminished. This observation specifies that in the composite wire, TOCNF decomposes at a lower temperature than α-DECHNs.

While learning the role of corn oil on gelatin-based nanocomposite membranes comprising nanochitin, differential scanning calorimetery (DSC) under nitrogen atmosphere was adopted by Sahraee et al. to inspect the thermal properties of emulsion nanocomposite films [82]. Incorporation of CNF to gelatin film increased T_m_ and ∆H_m_ of nanocomposite films which can be justified considering good compatibility and filling property of nanoparticles with gelatin. The melting point of nanocomposite gelatin films containing CNF (~102 °C) was found to be higher than that of net gelatin films (~83 °C). Another reason proposed for the fortifying effect of CNF was that it could increase the overall crystallinity of polymer, leading to higher transition temperature and enthalpy. Li et al. [83] researched chitin nanowhisker/metal ion (Zn^2+^) dual reinforcements in synthetic polyacrylamide (PAAm) network-based nanocomposite hydrogels; the thermal properties of the samples were measured using TGA under a nitrogen atmosphere. They observed that the nanocomposite hydrogels of PAAm/CNWs and PAAm/CNW/Zn^2+^ followed a degradation trend similar to that of the PAAm hydrogel, but the nanocomposite hydrogels showed higher residual weight in the thermograms. Additionally, the increase of the residual weight was observed in the PAAm/CNWs/Zn^2+^ hydrogel in comparison with the PAAm/CNWs hydrogel. These findings indicated that the presence of strong non-covalent interactions of CNWs with PAAm and Zn^2+^ enhanced the thermal stability of resultant nanocomposites.

### 5.5. Rheological Properties of Nanochitin-Based Composites

Rheology is an active and noteworthy method with which to comprehend the microstructures of biopolymers and their dispersions in solution or under fluid state, as impacted by the processing approaches; and afterward, to launch the optimum techniques and settings to understand the intricate melting and flow behaviors (e.g., viscoelastic properties), and to accomplish certain properties for the ultimate products. Rheology, as a complementary technique, offers evidence about the connections among fillers and how these can be altered with polymers. Mostly, rheological properties, viz., viscosity and yield stress, and the surface forces verified via examination procedures such as AFM, are nicely associated [87]. Studies have proven that interfacial, rather than bulk rheological factors can be made use of in envisaging the outcomes of the e-spinning procedure [88]. The bulk parameters are calculated by polymer content and directly influence jet instigation, whereas the interfacial parameters govern the durability of the jet and fiber construction [89]. Rheological properties of substantial importance are the linear viscoelastic moduli, G′ (storage modulus) and G′′ (loss modulus), the shear-rate-dependent viscosity and the yield stress. The linear viscoelastic moduli are reliant on the unperturbed (quiescent) microstructure, although the nonlinear rheological properties such as the viscosity and yield stress necessitate definite attention to flow effects on the microstructure [87].

Oscillatory analyses are generally attained with the help of controlled stress rheometers by means of only as much compression as required to offer highest contact area and least slip. Amplitude sweeps are initially completed so as to determine the linear viscoelastic range (LVR). Storage (G′) and loss (G″) shear moduli along with strain are recorded as afunctions of stress, at a fixed frequency (usually of 1 Hz). The constant strain value, at which the subsequent frequency sweeps are carried out to acquire the mechanical spectra, is then selected from the originally determined LVR for individual samples. Each mechanical spectrum is then documented at this constant strain value in the LVR. G′ and G′′, along with the tangent of the phase angle (tan *δ* = G″/G′) and complex viscosity (*η* *), are recorded as functions of increasing angular frequency (*ω*; rad s^−1^), after attaining steady-state for each point. On the whole, G′ exhibits a tendency to remain independent of frequency, and stays superior to G′′ in all the cases where the elastic component of the material is superior to the viscous component, archetypal of a gel-like character. The complex viscosity of the mixtures diminishes just about linearly with the augmentation of the frequency displaying shear thinning behavior, possibly owing to the structure of the hybrid polymer network [90].

Li et al. while studying the role of chitin nanowhisker and metal ion (Zn^2+^) dual reinforcements in synthetic polyacrylamide network-based nanocomposite hydrogels observed that as the CNW dosage in the blend was increased from 0.1% to 0.5%, the compression strength of the nanocomposites with constant Zn^2+^ concentration reached 6.95 ± 0.20 MPa, up from 1.95 ± 0.14 MPa [83]. The compression strength of the nanocomposites declined slightly when the CNWs’ concentration surpassed 0.5%, and the PAAm-CNW-0.5-Zn^2+^ hydrogel exhibited brilliant compression performance. They also assessed that for all hydrogels, the G′ values are always much larger than the corresponding G′′ values over the whole frequency range, indicating dominant elastic solid-like behavior. Among them, the PAAm-CNW-0.5-Zn^2+^ hydrogel had pronouncedly high storage/loss moduli due to the strong hydrogen-bonding and metal-coordination interactions within the network.

**Figure 18:**
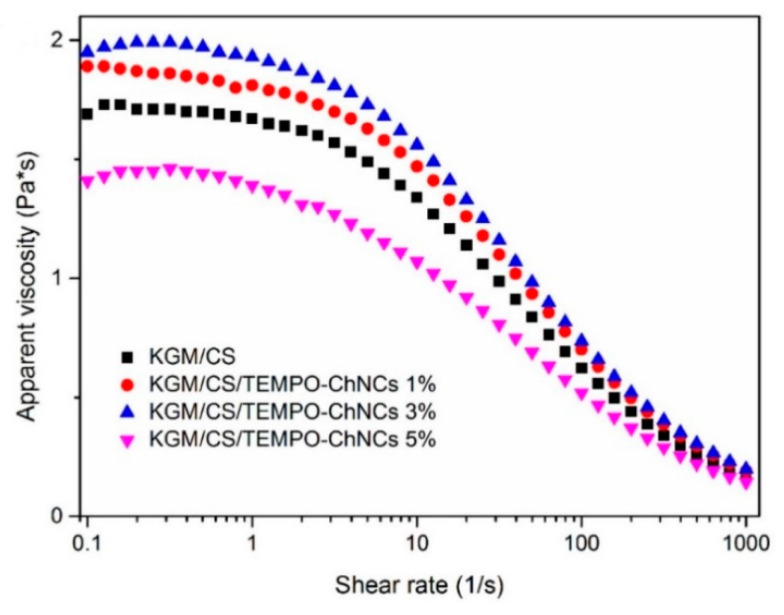
Steady rheological behavior of the obtained film forming solutions (FFSs) [86].

Bionanocomposite films based on konjac glucomannan (KGM), chitosan (CS) and TEMPO-oxidized chitin nanocrystals (CNCs) were fabricated by Sun et al.; they characterized the film forming solutions (FFSs) for their rheological properties since they play a huge role in determining the uniformity of the liquid coating layer, the mechanical properties and the spreadability [86]. The viscosity of all FFSs diminished as the shear rate increased, as shown in Figure 18, indicating the existence of the pseudoplastic properties or the shear thinning area of these FFS. (In the figure CNCs are represented as ChNCs).

**Figure 19:**
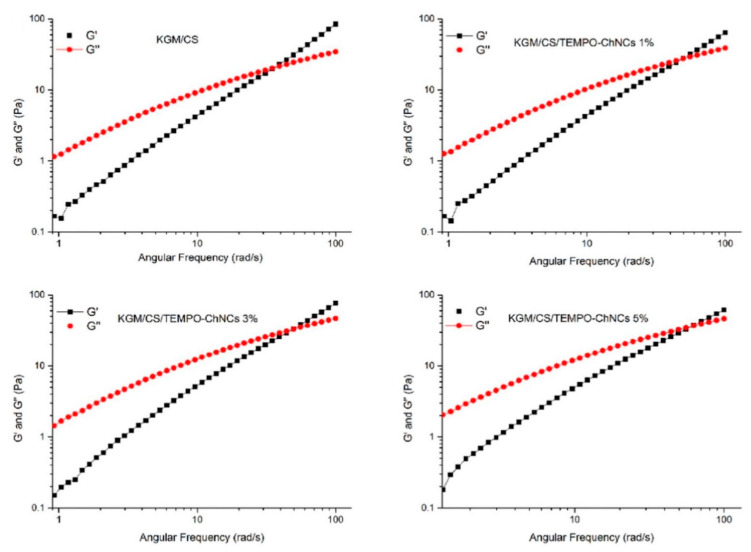
Dynamic rheological behavior of the obtained FFS [86].

The viscosity of the solution steadily improved as the incorporation of the TEMPO-CNCs increased from 1% to 3% compared with the KGM/CS FFS. These results were ascribed to the formation of novel hydrogen bonds and the destruction of their intermolecular interactions among KGM, CS and TEMPO-CNCs. Illustrating the dynamic rheological properties of the film forming solutions is significant for defining their viscous and elastic properties. Compared to the KGM/CS FFS, the values of G′ and G′′ increased as the concentration of the TEMPO-CNCs increased, as shown in Figure 19.

The incorporation of TEMPO-CNCs seemed to make the FSS microstructure better, thereby augmenting the mechanical properties of the resultant bionanocomposite films by establishing new chemical bonds and a vastly entangled network among KGM, CS and TEMPO-CNCs. The outcomes showed that the TEMPO-CNCs could improve the rheological properties of the FFS, and the integration of 3% (*w*/*w*) TEMPO-CNCs was proven to be the optimal concentration. Chitin whiskers are suitable to be incorporated as nanofillers in reinforcing polymer nanocomposites due to their high modulus values. Grafted samples present a significant increase in viscosity at high concentration values, without formation of the desired chiral nematic phase [87]. The role of varying nanochitin concentrations (0.25–1.0%, *w*/*v*) on the rheological properties, structure, compression behavior, pH and NaCl sensitivity of the gelatin hydrogels were methodically inspected by Li et al. by means of dynamic rheological experiments and compression assessments [91]. Dynamic rheological measurements of the nanocomposite hydrogels were carried out as functions of frequency sweeps, temperature sweeps and time sweeps, thereby proving that gelatin composite hydrogels showed excellent stability under conditions of increased acidity and high ionic strength with the incorporation of nanochitin whiskers (CHW). It was thus concluded that gelatin is capable of forming strong hydrogels with nanochitin in a saline environment.

**Figure 20:**
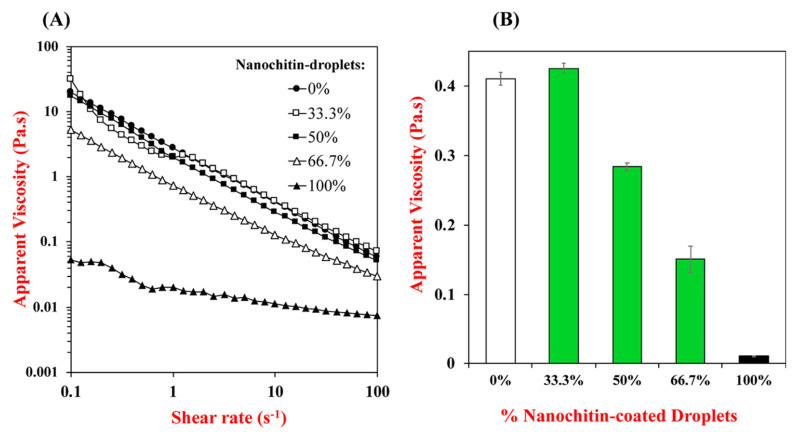
Impact of mixing ratio on the viscosity of mixed nanocellulose and nanochitin-emulsions: (**A**) apparent viscosity versus shear rate; (**B**) apparent viscosity versus percentage of cationic nanochitin-coated droplets present (shear rate = 10 s^−1^) [92].

Zhou et al. prepared mixed Pickering emulsions by blending anionic nanocellulose-stabilized lipid droplets with cationic nanochitin-stabilized lipid droplets, whose rheological properties were characterized by dynamic shear rheometry [92] (Figure 20). 

**Figure 21:**
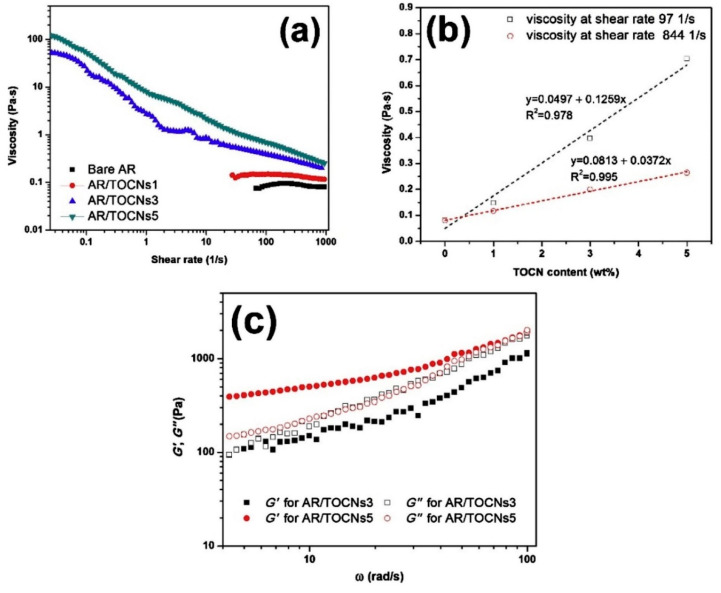
(**a**) Shear viscosity as a function of shear rate; (**b**) shear viscosity as a function of TOCN contents at two constant shear rates; (**c**) storage modulus G′ and loss modulus G′′ as functions of angular frequency *ω* [93].

They successfully proved that pickering emulsions with different rheological characteristics could be prepared by blending positive and negative droplets together in different ratios. While learning the effects of chitin nanocrystals in supple packing layers, Zhong et al. described that the TEMPO oxidised chitin nanocrystals (TOCNs) transformed the flow behavior of the water-based acrylic resin (AR) [93]. As displayed in Figure 21a, both the bare AR formulation and the AR formulation with low TOCN content (1 wt %) revealed almost Newtonian behavior, where the viscosity stayed practically unaffected with changing shear rates.

A more rigid network structure formed at the higher TOCN content (3 or 5 wt %) led to a distinctive shear thinning flow behavior; with higher shear rate, the viscosity declined since the network was disturbed. Figure 21b shows that the AR viscosity increased linearly at medium or high constant shear levels with a higher TOCN concentration; the effect of the material at the higher constant shear rate was decreased. The dynamic viscoelastic nature of the water-based AR was also reformed with the integration of TOCNs. Figure 21c shows the storage modulus G′ and loss modulus G′′ of the AR/TOCNs3 and AR/TOCNs5 formulations. The AR/TOCNs5 formulation exhibited solid-like (elastic) behavior at low angular frequencies as evidenced by the G′ > G′′. It also had a higher storage modulus along with the whole angular frequency range because of its higher TOCN content. The outcome shows that the TOCNs have the capability for modifying the rheological and mechanical properties of water-based coating arrangements. Chitin hybrid biodegradable materials reinforced with graphene oxide nanosheets (nGO) were prepared and characterized by Gonzalez et al. [90], where nGO acted as a filler, inducing structural rearrangements in chitin with the occurrence of new hydrogen bonds among the chains. Results proved that rheological behavior of the material became more solid-like with increasing nGO content. A physical composite nanochitin/microemulsion hydrogel was explored by Wang et al. for an extended release of hydrophobic compounds (drugs) under in vitro physiological surroundings, whereby it was made known that the composite hydrogels displayed slightly inferior values of storage modulus and loss modulus when compared to pure nanochitin hydrogels [94]. However, the G′ values of all the composite hydrogels were roughly around 1000 Pa. Chemical crosslinking studies of glutaraldehyde (Glu) with nanochitin were carried out by Liu et al., which proved that G′ values for nanochitin (x)/Glu (0.4) hydrogels were at least an order of magnitude higher than for the sturdiest chitin-derived hydrogels published hitherto [95].

### 5.6. Barrier Properties of Nanochitin-Based Composites

Evaluation of barrier properties for composites is important due to the role of water in the production of microbial and the degradation results [57]. A coating or a film is very often needed in product packaging to minimize or prevent the transfer of moisture between the product and the surrounding environment. Water vapor permeability is the most imperative and extensive properties of bio-based polymer films, due to the direct influence on the deteriorating reactions in packaged food products. The qualities of the membrane will usually be strengthened by using appropriate additives [96]. In bio-polymer films, a broad variety of nanofillers have been applied to enhance the thermal and mechanical properties, as discussed before, and also water vapor barrier properties. Sustainable membrane structures dependent on nanomaterials produced from polysaccharides have drawn great deal of interest in recent times [93]. Chitin nanomaterials, together with cellulose nanomaterials, have garnered attention as products for safe gas barriers. Chitin is used as an origin of nanoparticles like those used in barrier films as one of the most important nanofiller reinforcements [97,98]. Nonetheless, the effects of crystallinity, charges and measurements of chitin nanomaterials on the efficiency of their oxygen barriers are poorly explored [99]. Nanoparticles, nanofibers and nanocomposites produced using chitin reflect new families of polymer carriers and matrices capable of improving film/composite mechanical and barrier properties [100]. The intent of a barrier layer or film in a packaging product is to decelerate or essentially eliminate the advance of oxygen, water vapor or other molecules, thereby increasing the shelf life, protection and likely also the taste of goods—especially in the case of foods. The nanomaterials can be predicted to swell with rising relative humidity owing to the ample quantity of water-loving hydroxyl groups found on all polysaccharides. It is therefore widely established that the capacity of polysaccharide-based films to withstand water vapor would decrease sharply with rising relative humidity. Many considerations such as the chemical makeup of sources, the introduction of plasticisers and the crystallinity of the polymer material often influence the water barrier role of polysaccharide membranes and coatings [98].

Permeability of gases through a substrate is described as the measurement of the transmission of permeate (gas and vapor) across a resistant medium. The predominant mode of gas distribution in a film without any flaws such as pinholes or cracks is the solubilization of gas molecules on a film surface (Henry’s law) accompanied by the diffusion across the mass (Fick’s law), and lastly desorption of gas particles from the opposing surface. There have been clear associations between the capacity of a film to inhibit the flow of oxygen gas and its ability to obstruct the passage of non-polar organic compounds in either the gaseous or the liquid state. The very same hydrogen bonds that bind the barrier film components together are often heavily affected by the influence of moisture or humidity [98]. Since film water vapor permeability (WVP) is based on the solubility and dissemination of particles throughout the film, nanoparticles may reduce this permeability by growing cross-linkage across polymer chains and loading the membrane porosity. Since nanoparticles can boost the cross-linking and cohesion of intermolecular interactions, they strengthen the oxygen barrier properties of the membranes [101].

The study discussed by Hubbe et al. demonstrates the role of chitin nanoparticles on barrier film efficiency, and that these environmentally friendly components are often chosen in the resulting films where one of the objectives is to induce antimicrobial effects [98]. The latest studies have revealed that nanochitin can strengthen the barriers of gelatin films to water vapor by loading properties and causing a circuitous course across water molecules [97,101,102]. Chitin nanoparticles extracted from different sources, irrespective of their types and modifications, have been proven to improve water, gas and light barrier properties of composite films [57,84,86,93,97,99,103]. The barrier efficiencies of recorded nano-chitin-reinforced films were assessed using different protocols. Among the different metrics addressed, it seems that the most demanding requirements include avoiding the permeation of both water vapor and oxygen, and also to continue to resist this permeation even as the film is subjected to a broad spectrum of moisture. The membranes must therefore be adequately crucial to prevent the simple passage of oxygen, water vapor, aromas or bacteria into or out of the food packet [98].

#### 5.6.1. Water/Water Vapor Permeability (WVP)

It has been reviewed that PLA-based nanostructured composites, comprising nano-fillers (such as phillosilicates, cellulose or chitin nanofibrils) can be capable of expanding the barrier properties of biodegradable composites without altering their optical properties (transparency). The attained Ag-non-woven tissue made of chitin nanofibers (CNF) and chitosan (CS) displayed the ability to diminish the burden bacteria of infested skin, and to augment the reparative capacity of the skin, presenting boosted antibacterial activity in conjunction with wound healing efficacy [100]. It has also been stated that particle concentrations influence the degree of coalescence during emulsification of solid-stabilized emulsions; actually, the coalescence can be decreased by rising the number of chitin nanoparticles in emulsions, which permits the establishment of links of accumulated units in the continuous phase [103]. Films fabricated by Ge et al. exhibited gradually decreased WVP values from 7.8 × 10^−4^ to 6.7 × 10^−4^ g day^−1^ m^−1^ atm^−1^, perhaps due to the intramolecular and intermolecular connections among black rice bran anthocyanins (BACNs), oxidized chitin nanocrystals (O-CNCs) and gelatin, which condensed the breach in the film [97]. In addition, the massive aromatic and pyrylium rings in the skeleton of anthocyanins could impede the internal networks of BACNs-Ch films and lessen their water vapor affinity. Even though nanochitin could decline WVPs of gelatin membranes, they still had high affinity to water vapor due to the hydrophilic character of gelatin and nanochitin.

**Figure 22:**
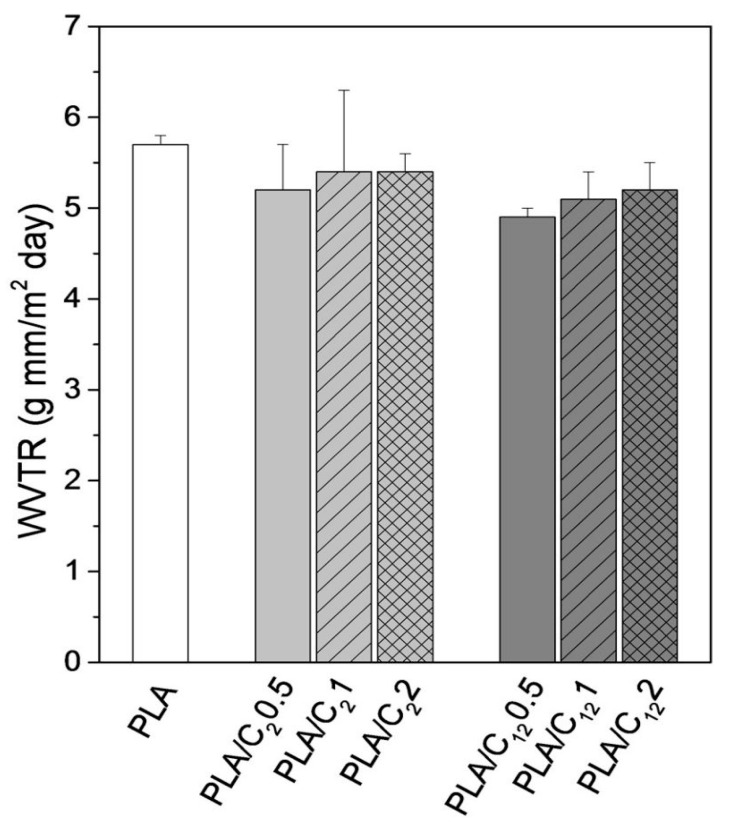
Water vapor transmission rates (WVTRs) of PLA films and PLA nanocomposites incorporated with nanochitin modified by acetic anhydride (C2) and fatty aliphatic chains (C12) [57].

Salaberria et al. reported the modification of chitin nanocrystals via acylation using anhydride acetic and dodecanoyl chloride acid to improve their compatibility with PLA [57]. A minor diminution in the water vapor transmission rate (WVTR) (Figure 22), could be observed which is directly connected with the integration of hydrophobic clusters on the exterior of the chitin nanocrystals (CNCs).

Both types of modified CNC appeared to be efficient components, i.e., they amended the hydrophobic nature of the PLA nanocomposite membranes to a small extent. However, the decline in the WVTR was more obvious for the PLA-based nanocomposites manufactured with nanochitin modified with fatty aliphatic chains (C_12_) rather than with acetic anhydride (C_2_) [57]. Reports have shown that in the case of carrageenan/chitin nanofibrils composites, moisture content of films pronouncedly diminished after combining with nanochitin [104]. The WVP of maize starch nanocomposite films declined with chitin nanowhisker (CNW) content increasing from 0 to 2%. Concerning starch/chitin nanocrystals (CNC) nanocomposite membranes, when 5 and 10 wt % of nanochitin were added, WVTR values were lowered. Along with the clustering nature of chitin, another reason for the meagre water vapor barrier property is the occurrence of high residual NH_2_ groups, which causes high attraction for water OH groups at the surfaces of the chitin nano-objects [96].

#### 5.6.2. Gas/Oxygen Permeability (OP)

**Figure 23:**
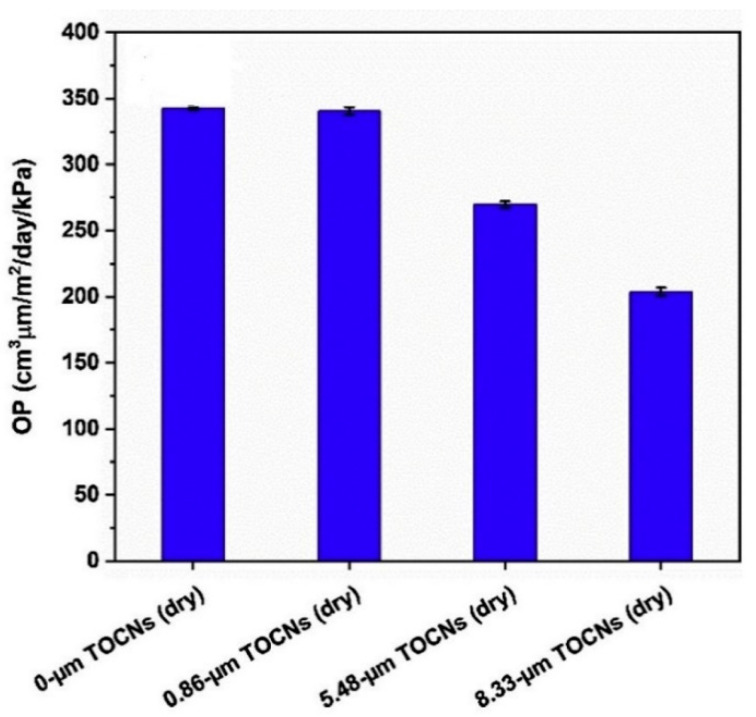
Oxygen permeability (OP) of BOPP (biaxially oriented polypropylene) laminates as a function of TOCN (TEMPO oxidized chitin nanocrystals) dry thickness [93].

Studies by Sahraee et al. have revealed that nanochitin assimilation in gelatin films significantly reduced their water vapor and oxygen permeability values, thereby helping them to preserve the peroxide value of cakes at lesser levels than other associated polymers [101]. At the same time, it has been recommended that applying gelatin emulsion film as the second layer could be pertinent. In another parallel research, Zhong et al. observed that TEMPO oxidised chitin nanocrystals (TOCNs) as a dispersed phase in the acrylic resin matrix did not augment the oxygen barrier property of the subsequent composites, but the neat continuous TOCN coating layer upgraded the oxygen barrier property of the laminates [93] (Figure 23). 

**Figure 24:**
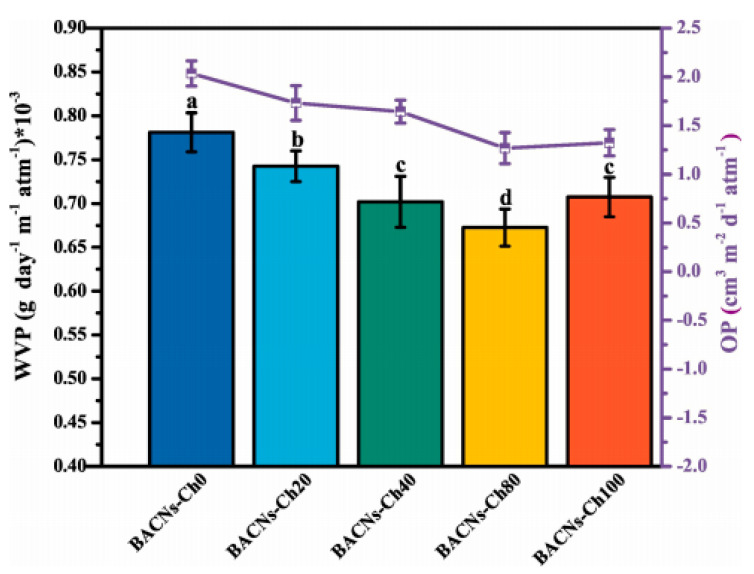
Water vapor permeability and oxygen permeability of BACNs-Ch films [97].

They have also proposed the use of a multilayer structure for grander oxygen barrier properties. Other studies have specified that nanochitin are capable barrier materials; even trace amounts or a thin layer added to different substrates could result in outstanding barrier properties [105,106,107]. Figure 24 illustrates the barrier properties of gelatin/oxidized chitin nanocrystal nanocomposite films reliant on black rice bran anthocyanin (BACN) content. 

With the rise of BACNs, the oxygen permeability (OP) exhibited an alterable waning from 2.035 cm^3^ m^−2^ d^−1^ atm^−1^ to 1.323 cm^3^ m^−2^ d^−1^ atm^−1^. Perhaps the hydrogen bond relations between BACNs and matrix controlled or protracted the penetrating path of oxygen. It was proposed the BACNs incorporation could pointedly boost the oxygen barrier. Remarkably, the OP of BACNs-Ch100 group was better than that of BACNs-Ch80 group, maybe due to the aggregation of anthocyanins and uneven distribution of matrix [97]. The drop in oxygen transmission rate (OTR) of PVA by various chitin nanomaterials were investigated by Tran et al. [99]. The virgin PVA film exhibited an OTR of 55.35 ± 9.22 mL/m^2^·day, which was inadequate for usage in most food-packaging applications. The incorporation of chitin nanomaterials enhanced the oxygen barrier properties of the PVA membrane, thus aiding it to achieve values comparable to that of ethylene vinyl alcohol (EVOH) (<5 mL/m^2^·day), an exemplary oxygen-barrier polymeric film. Conversely, graphene oxide, less considered in the food application arena than chitin has capable characteristics in the fabrication of food wraps owing to its oxygen barrier nature. Furthermore, the chemical groups in GO sheets unlocks the application fields for chitin, a low reactive polysaccharide [90].

### 5.7. Optical Properties

**Figure 25:**
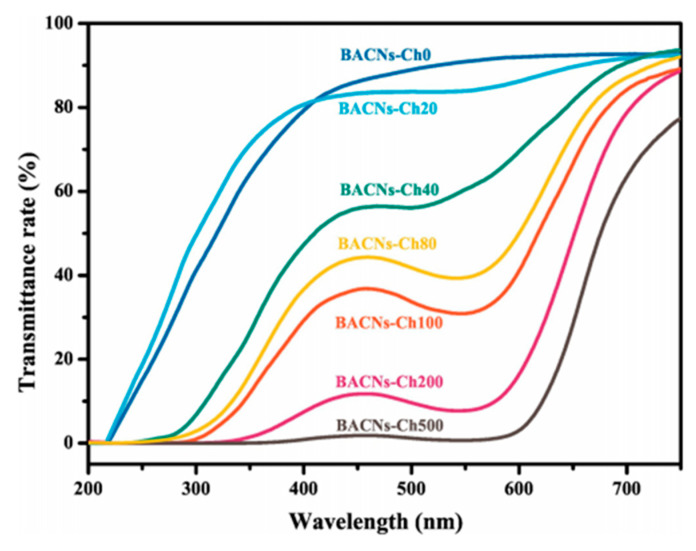
Light transmission rates of the BACNs-Ch films containing different BACN concentration [97].

Studies revealed that the use of nanochitin in versatile polypropylene films, either as an additive in the framework or as a continuous layer in a laminate, would not affect the initial packaging’s optical clarity; this was an added advantage as transparency is a desirable feature in modern food packaging. Shankar et al. studied the apparent color and clarity of carrageenan/chitin nanofibril films defined by the Hunter Lab-values and the percent visible light transmittance (660 nm), respectively. It could be seen that lightness (*L*-value) and transmittance of the composite films reduced linearly, while greenness (*a*-value), yellowness (*b*-value), and total color difference (ΔE) of the composite films improved monotonously with upsurge in the concentration of chitin nanofibrils [104]. Figure 25 demonstrates the light transmission spectra for the gelatin/black rice bran anthocyanins (BACNs)/oxidized chitin nanocrystals (O-CNCs) films developed by Ge et al. [97]. 

The light transmittance rate at 560 nm declined from 91.45% to 31.27% as the BACN contents were augmented from 0 mg to 100 mg, which was attributed to the merging effect of BACNs on the well-organized construction of O-CNCs and gelatin. The subsequent figures verified that the films comprising of BACNs could defend ultraviolet light and diminish the food decay instigated by ultraviolet light and explicit range of visible light (200–400 nm).

## 6. Applications of Nanochitin

New areas in the use of chitin have evolved across a variety of purposes including nanocomposite materials, electrical and electronic devices, cosmetics, agriculture, packaging, environmental and biomedical applications. Nanochitin serves as a possible nanofiller to be used as chitin nanocrystals, oxidized-chitin nanocrystals, nanofibrils, partly deacetylated chitin nanofibers, etc. to stabilize biopolymer-based composites [84]. The use of polymers and nanochitin to produce bio nanocomposites offers a good opportunity to prepare bio plastic materials with enhanced functional and structural properties. Agricultural scientists have analyzed nanochitin for their effects on crop growth enhancement, metabolism of carbon or nitrogen, stress tolerance, and elicitation of plant disease response [108,109,110]. Effects of nanochitin on improving grain yields and winter wheat quality were recorded by Xue et al. where they showed that 0.006 g kg^–1^ of nanochitin in soil could pointedly boost the yield by 23% for multi-spike wheat (MSW) and 33.4% for large spike wheat (LSW), with substantial rises of net photosynthesis rate, stomatal conductance, intercellular CO_2_ concentrations, and transpiration rate in flag leaf at the grain filling stage [111]. Cheng et al., while studying the motivating effect of chitin nanoparticles on the metabolism of carbon and nitrogen, and the enhancement of grain yield and crude protein in winter wheat, suggested that nanochitin encouraged nitrogen metabolism more than carbon metabolism and increased crude protein concentration in grain by 13.26% and grain yield by 27.56% [112]. When applied at an optimal rate, cationic nanochitin whisker could inhibit root rot tobacco and wheat diseases, promote photosynthesis, and enhance the grain yield of winter wheat by promoting net photosynthesis rate, stomatal conductivity, intercellular CO_2_ concentrations, and grain-filling transpiration rate in flag leaf. Grain protein, iron, and zinc in wheat also increased substantially when treated with nanochitin. Liang et al. researched nanochitin whisker’s antifungal activity against crown rot wheat diseases and proved to have important inhibitory effects on mycelial growth and conidial production [113]. This suggested that nanochitin has good antifungal activity against soil-borne wheat pathogens and decreases the use of chemical fungicide in wheat planting. The impact of nanochitin suspensions on tobacco seed germination, seedling proliferation and symbiotic interaction with fungicides were investigated by Zhou et al. in indoor and field trials, while evaluating the bioactivity of nanochitin on tobacco [114]. Researchers found that 0.004% (*w*/*v*) of nanochitin enhanced tobacco seed germination and considerably reduced the mean time for germination; 0.005% (*w*/*v*) of nanochitin increased tobacco stem length, stem girth, leaf number and leaf area, and 0.001% (*w*/*v*) of nanochitin had simulatory effects on tobacco root rot inhibition when mixed with metalaxyl mancozeb and thiophanate methyl fungicides. This suggests that suspensions of nanochitin have a huge potential to safeguard tobacco from root rot diseases and to minimize the need for toxic fungicides in tobacco fields. Farmers have made significant use of nanochitin and its derivatives as biopesticides, biofertilizers and as agricultural film in seed and fruit coating among the vast array of alternative products for agricultural purposes identified so far.

Chitin nanofibrils (CNFs), consisting of colloidal nano-rods, institute the crystalline fraction of chitin isolated from marine food waste; they have been documented to exhibit anti-microbial properties and promote regeneration of cells. Chitin nanowhiskers also fall in the category of cost-effective fillers that can impart antibacterial activity for applications involving wound healing and food packaging. The antibacterial activity of chitin nano whiskers was explored Jiang et al., where lysozyme was adsorbed onto the surface of nanowhiskers which exhibited enhanced antibacterial activity when compared to lysozyme alone [115]. Chitin nanowhiskers extracted from crab shells were also used to prepare composite films in combination with maize starch. The films showed excellent antibacterial effect against gram positive bacteria but not against gram negative *E. coli.* The films also showed improved mechanical strength than neat starch films.

Nanochitin has been extensively studied in multifarious biomedical applications involving tissue engineering, drug delivery, wound healing etc. Torres-Rendon et al. manufactured bioactive gyroid scaffolds developed by sacrificial templating of nanocellulose and nanochitin hydrogels as edifying models for biomimetic tissue engineering, based on prior findings that human fibroblast adhesion to low deacetylation chitin is weak whereas human fibroblast and keratinocyte adhesion to highly deacetylated chitin is high [116]. Wang et al. effectively produced a physical composite nanochitin/microemulsion hydrogel for extended release under in vitro physiological conditions of hydrophobic compounds (drugs) [94]. They demonstrated that the composite hydrogel successfully embedded Nile Red in phosphate-buffered saline with an extended release time of 60 h (PBS pH 7.4, equivalent to biological conditions). The wound healing abilities of superficially deacetylated nanofibrils of chitin (SDACNFs) have been tested by Izumi et al.; they showed that SDACNFs successfully mediated re-epithelium, and fibroblast and collagen tissue proliferation [117]. Nano chitin was also used as an alternate natural nanomaterial in a study performed by Tang et al. to blend with cellulose fibers to make high-strength paper which improved the colorimetric output of glucose bioassays [118].

**Figure 26:**
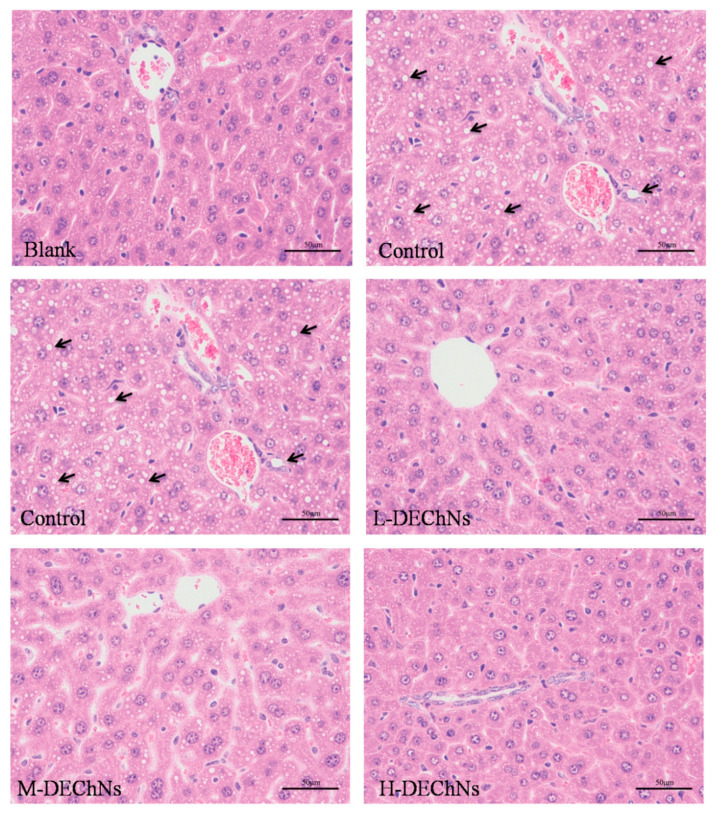
Histological examination of liver tissues from mice in the blank group (fed a normal diet and saline solution); control group (fed a high-fat diet and dilute acetic acid solution); and the L, M and H-DEChN groups (fed a high-fat diet and low, medium, high doses of DEChN dispersions, respectively). The arrows point out the fat vacuole in the cytoplasm [120].

Chitin nanofibers and surface deacetylated chitin nanofibers have been reported as potential functional foods for patients having obesity [119]. The beneficial role of deacylated chitin nanofibers (DEChNs) in reducing hypercholesterolemia was investigated in mice by dividing them into five groups [120]. The blank group comprised mice fed with a normal diet and saline solution; the control group included mice fed a high fat diet and dilute acetic acid solution; and remaining three groups of mice were treated with different doses of DEChNs. The histopathology studies of liver revealed that the control group showed a large number of fat vacuoles (Figure 26). The blank group was diploid for vacuole formation, and that was the case for the mice treated with DEChNs too. The study clearly showed that the chitin nanofibers could effectively control lipid accumulation and could prevent fatty liver formation.

Chitin nanofibrils have been majorly applied as nanofillers in the reinforcement of both natural and synthetic composites due to their size, mechanical strength and relevant biological properties. The various applications of chitin-based composites have been listed in Table 1.

Sahraee et al. fabricated films for cake packaging based on bovine gelatin-nanochitin-nano ZnO [101]; such films are suitable for packing of sponge cakes that do not involve preservatives because this packaging can avoid fungal growth for longer periods of time and could preserve the chemical and organoleptic consistency of cakes even more. Nano chitin composites are also employed for managing the freshness of food products, especially sea foods. Although Wu et al. effectively prepared intelligent chitosan-based films containing black rice bran anthocyanins for tracking seafood and animal-based protein contamination, they noted that the introduction of oxidized chitin nanocrystals improved the film’s mechanical and barrier properties [121]. Analogous research was recorded by Ge et al. in which gelatin/oxidized chitin nanocrystal composite films comprising black rice bran anthocyanins were used for related applications for freshness monitoring [97]. Such films were pH-sensitive and displayed impressive improvements in color in different buffer solutions that could be used by noticeable color changes to track the freshness of shrimp.

Nano scale chitin and its derivatives also play extensive roles in environmental applications. Dye adsorption sectors and water treatment sectors have made use of the superior absorption capacities of chitin nanoparticles. One of the many such studies include the report by Gopi et al. where the researchers produced and examined shrimp shell chitin nano whiskers (CNW) for improved crystal violet adsorption [125]. A series of adsorbent materials such as chitin-Fe_3_O_4_, cellulose, and cellulose-Fe_3_O_4_, along with CNW, have been used to compare the results and to better explain adsorption phenomena. When compared to various sorbent materials, the newly synthesized chitin nanowhiskers exhibited augmented efficiency of removal (79.13%) and adsorption potential (39.56 mg g^−1^). The results showed that CNW could be a viable candidate for the elimination of crystal violet from polluted water. As a strong-efficiency adsorbent for water treatment, biohybrid hydrogel and aerogel from self-assembled nanocellulose and nanochitin were manufactured by Zhang et al. [60]. Biohybrid aerogels (BHA) demonstrated super-high adsorption potential of 217 mg·g^–1^ for As(III) under neutral pH conditions and 531 mg·g^–1^ for Methylene Blue (MB) under an alkaline aqueous atmosphere with accelerated kinetics of adsorption, in striking contrast to traditional biobased adsorption. In fact, the BHA is renewable, which still demonstrated a strong 505 mg·g^–1^ MB adsorption efficiency even after five consecutive cycles of adsorption–desorption. In parallel reported literature, Wu and colleagues developed biodegradable chitosan hydrogel beads containing nanochitin for the rapid and effective elimination of Cu(II) and magnetic chitosan microfibers comprising of nanochitin via continuous injection gelation method for removal of Ni(II) ions from aqueous solutions [126,127]. The Fe_3_O_4_ encapsulated polystyrene (FP) microparticles (as a magnetic separation material) and nanochitin (n-CT, as an advantageous natural metal-cation adsorbent) were integrated in situ into poly(vinyl alcohol)-enhanced chitosan hybrids (PVA/CTS) to form environmental-friendly nanocomposite hydrogels (FPCC) with magnetic segregation capability. Relative to the pure PVA/CTS hydrogels with a Cu (II) adsorption value of 38.7 mg/g, the FPCC hybridized hydrogels provided around 1.7-fold Cu (II) adsorption efficiency. Within 10 h, the FPCC magnetic microfibers (adsorbent dosage: 1 g, pH: 4.1, and temperature: 293 K) could adsorb 99.7% Ni (II), after which the hybridized microfibers were shown to be readily isolated from the aqueous solution by a magnetic separation process. The manufacture of nanochitin/manganese oxide-biodegradable composite adsorbent for heavy metal ions was also documented by Krivoshapkin et al. where it was found that the use of organomineral composite sorbents adopted the Ni^2+^ > Cu^2+^ > Sr^2+^ pattern for removal of heavy metal ions, with a sorption efficiency of 114.0 ± 1.1 mg/g for Ni^2+^ [122]. The harvested biodegradable sorbents were aimed at addressing ecological problems correlated with radioactive metallic ions polluting natural water.

Yet another field that nanochitin finds application includes electrical and electronic devices. For example, biobased cryogel membranes were implemented as electrolyte holders in dye solar cells (DSC) [128], thus facilitating carrier transport during service. It was also observed that the system performance and stability were also improved with the help of chitin nano fibers. Conductive biomass-based composite wires with cross-linking anionic nanocellulose and cationic nanochitin as scaffolds were developed by Xu et al., who efficiently designed a collection of conductive composite wires by coupling dispersions of multi-wall carbon nanotubes (MWCNTs) and TEMPO-oxidized cellulose nanofibers (TOCNFs) with specific MWCNT compositions into a dispersion of partly deacetylated α-chitin nanofibers (α-DECHNs) proceeded with a drying procedure [85]. When the content of MWCNTs exceeded 14 wt %, the designed composite wire could illuminate LED at 5 V voltage, demonstrating the enormous latency of this product based on biomass in conductive material deployment.

## 7. Conclusions and Future Perspectives

Management of crustacean shell waste has become a huge problem in the sea food industries. The effective processing of shell waste from marine organisms is being exploited for extracting chitin and its derivatives. In contrast to synthetic polymers, natural polymers such as chitin are good raw materials for producing inexpensive, rapidly degradable bioplastics. Despite the described advantages, the utilization of biopolymers has been limited due to concerns regarding their poor mechanical and barrier properties, which can be improved by adding bio-based reinforcing agents (fillers), forming composites. Nanochitin have shown considerable advantages in various sectors, such as biomedicine, the food industry and agriculture, due to its unique properties. Future research should focus on finding greener solutions for cost effective processing of nanochitin. Use of eco-friendly solvents or recycling of the reagents can minimize the toxicity due to hazardous effluents. A combination of enzymatic and chemical techniques for the isolation of chitin can also be considered. However, a fundamental understanding of structure–function relationship is essential for the development of multifunctional composites that could probably overcome the current constraints.

## Figures and Tables

**Table 1:** Recent applications of chitin nanocomposites.

Form (Whiskers/Crystals/Fibrils)	Matrix/Filler	Form of Composites (Hydrogels/Films/Aerogels)	Applications	References
Nanowhiskers	Cellulose	Films	High strength paper for bioassay applications	[118]
Nanocrystals	Water-based acrylic resin (WBAR), Biaxially oriented polypropylene (BOPP)	Films	Flexible packaging coatings	[93]
Nanocrystals	Carboxymethyl cellulose	Films	Active food packaging materials and biomedical applications	[84]
Partly deacetylated chitin nanofibre (PDCNF)	TEMPO-oxidized cellulose nanofibre (TOCNF)	Hydrogels and aerogels	Green renewable high-efficiency adsorbent for water purification	[60]
Oxidised chitin nanocrystals	Chitosan	Films	Seafood spoilage monitoring	[121]
Nanocrystals	Manganese oxide	Powder	Hybrid sorbent for heavy metal ions	[122]
Partially deacetylated α-chitin nanofibers (α-DECHNs)	TEMPO-oxidized cellulose nanofibers (TOCNFs)	Wires	Conductive material applications	[85]
Oxidised chitin nanocrystals	Gelatin	Films	Fish freshness monitoring	[97]
Surface-deacetylated chitin nanofibers	Sacran polysaccharide	Freeze-dried pellets	Extended-release excipient for tetrahydrocurcumin (THC) for wound healing	[123]
Nanofibers	Cellulose nanocrystals (CNC)	Spray coating	Enhancement of oxygen permeability of PLA films	[107]
Nanofibers	Pectin + Nanolignocellulose		Improvement of probiotic survival in fruit juice and under gastrointestinal conditions	[124]
Nanofibrils (Surface-deacetylated cationic NFs)	Cellulose nanofibrils	Hydrogels	Biomimetic scaffolds for bone tissue engineering	[116]
